# *FoxO6* regulates Hippo signaling and growth of the craniofacial complex

**DOI:** 10.1371/journal.pgen.1007675

**Published:** 2018-10-04

**Authors:** Zhao Sun, Clarissa S. G. da Fontoura, Myriam Moreno, Nathan E. Holton, Mason Sweat, Yan Sweat, Myoung Keun Lee, Jed Arbon, Felicitas B. Bidlack, Daniel R. Thedens, Peggy Nopoulos, Huojun Cao, Steven Eliason, Seth M. Weinberg, James F. Martin, Lina Moreno-Uribe, Brad A. Amendt

**Affiliations:** 1 Department of Anatomy and Cell Biology, and the Craniofacial Anomalies Research Center, Carver College of Medicine, The University of Iowa, Iowa City, IA, United States of America; 2 Iowa Institute for Oral Health Research, College of Dentistry, The University of Iowa, Iowa City, IA, United States of America; 3 Department of Orthodontics, College of Dentistry, The University of Iowa, Iowa City, IA, United States of America; 4 Department of Oral Biology, School of Dental Medicine, University of Pittsburgh, Pittsburgh PA, United States of America; 5 Private practice, Cary, North Carolina United States of America; 6 The Forsyth Institute, Cambridge, MA United States of America; 7 Department of Psychiatry, Carver College of Medicine, The University of Iowa, Iowa City, IA, United States of America; 8 Department of Physiology, Baylor College of Medicine, Houston, TX, United States of America; Max Planck Institute for Molecular Genetics, GERMANY

## Abstract

The mechanisms that regulate post-natal growth of the craniofacial complex and that ultimately determine the size and shape of our faces are not well understood. Hippo signaling is a general mechanism to control tissue growth and organ size, and although it is known that Hippo signaling functions in neural crest specification and patterning during embryogenesis and before birth, its specific role in postnatal craniofacial growth remains elusive. We have identified the transcription factor FoxO6 as an activator of Hippo signaling regulating neonatal growth of the face. During late stages of mouse development, FoxO6 is expressed specifically in craniofacial tissues and *FoxO6*^*-/-*^ mice undergo expansion of the face, frontal cortex, olfactory component and skull. Enlargement of the mandible and maxilla and lengthening of the incisors in *FoxO6*^*-/-*^ mice are associated with increases in cell proliferation. *In vitro* and *in vivo* studies demonstrated that FoxO6 activates *Lats1* expression, thereby increasing Yap phosphorylation and activation of Hippo signaling. *FoxO6*^*-/-*^ mice have significantly reduced Hippo Signaling caused by a decrease in *Lats1* expression and decreases in *Shh* and *Runx2* expression, suggesting that *Shh* and *Runx2* are also linked to Hippo signaling. In vitro, FoxO6 activates Hippo reporter constructs and regulates cell proliferation. Furthermore PITX2, a regulator of Hippo signaling is associated with Axenfeld-Rieger Syndrome causing a flattened midface and we show that PITX2 activates *FoxO6* expression. Craniofacial specific expression of FoxO6 postnatally regulates Hippo signaling and cell proliferation. Together, these results identify a FoxO6-Hippo regulatory pathway that controls skull growth, odontogenesis and face morphology.

## Author summary

The basic question of how human faces develop, undergo morphogenesis and grow after birth to define our final characteristic shape has been studied from the earliest days of comparative vertebrate developmental research. While many studies have shown the factors and mechanisms that contribute to the cells and tissues of the face during embryology, fewer studies have determined mechanisms that promote face growth after birth and into childhood. In our quest to understand developmental mechanisms of facial growth we used murine gene expression and bioinformatics analyses combined with human 3D facial variations and genome-wide association studies to identify genes and variants controlling post-natal face growth. Bioinformatics analyses of mouse craniofacial gene expression identified FoxO6 as a transcription factor expressed at late stages of face development. Ablation of *FoxO6* in the mouse resulted in specific anterior growth of the mouse face. The increased *FOXO6* expression activated Hippo signaling to reduce face growth. These data indicate that changes in *FOXO6* expression control face growth during early childhood.

## Introduction

Hippo signaling is a major determinant in regulating organ size and tissue regeneration. Several lines of evidence indicate that developing organs possess intrinsic mechanisms that modulate their final size [1, 2]. Genetic studies have established that the Hippo pathway plays a crucial role in organ size, controlling cell number by modulating cell proliferation and apoptosis [3–8]. This pathway is triggered by the binding of extracellular ligands, which activate Mst1/2. Active Mst1/2 phosphorylates the Lats1/2 kinase [9], which is in turn activated, and subsequently phosphorylates and inactivates the Yes-associated protein (YAP) 1 transcriptional co-activator, a major downstream effector of the mammalian Hippo pathway [9–11] and TAZ, causing them to accumulate in the cytoplasm [12–14]. Therefore, upon activation of Lats1/2, the expression of target genes related to cell survival are inhibited due to the retention of YAP and TAZ in the cytoplasm. In contrast, the unphosphorylated (i.e. active) forms of YAP and TAZ associate with transcription factors (TFs) of the TEAD/TEF family in the nucleus, activating the expression of target genes, and thereby promoting cell proliferation and inhibiting apoptosis [8, 15, 16]. Thus, Hippo signaling represses cell proliferation and stimulates apoptosis. The role of Hippo signaling in craniofacial development was recently shown through the inactivation of Yap and Taz in early neural crest-derived structures of the craniofacial complex [17, 18]. This study identified how Hippo signaling was involved in neural crest specification and patterning during embryogenesis and before birth. However, the transcriptional mechanisms that regulate Hippo signaling components in postnatal craniofacial development are not well understood. We have identified a new transcriptional regulator of Hippo signaling specifically expressed in the craniofacial complex.

The molecular mechanisms that control species-specific craniofacial growth and give rise to the different vertebrate head sizes and morphology include signaling and growth factors [19–31]. In particular, the Wnt, Fgf, Bmp, Shh, and Tgf-β signaling pathways control the early patterning and growth of the craniofacial skeleton by regulating the migration, proliferation, differentiation and transformation of cells derived from the mesoderm and cranial neural crest [32–41]. These factors and pathways interact and intersect to control development of the brain and skull [21, 22, 25, 26, 40, 42–44]. Tissue-tissue interactions that give rise to cell fate decisions are fundamental to the development of head structures, especially for the patterning and morphogenesis of craniofacial structures [45–47]. These early developmental cues drive the morphogenesis and patterning of perinatal craniofacial tissues. However, regulation of postnatal craniofacial growth is less well known, and although members of the early developmental pathways above are likely implicated, other less well-known factors could also play significant roles in determining adult head size and shape. Continued growth of the head and face after birth are critical for healthy ontogeny. Yet, it has been unclear how Hippo signaling affects these growth and expansion processes of the craniofacial complex after birth. We provide evidence that Hippo signaling components regulate this postnatal growth, which is distinct from early patterning events.

FoxO6 is a TF that contains a Forkhead (winged helix) domain and is encoded by one of the *FoxO* class genes [48, 49]. Mammals have four *FoxO* members (*FoxO1*, *FoxO3*, *FoxO4* and *FoxO6*) [50]. In humans, single nucleotide polymorphisms in *FOXO1* and *FOXO3* have been associated with increased longevity and in invertebrates the expression of *FoxO* proteins can increase their life span [51–55]. *FoxO6* is the most recently identified *FoxO*-encoding gene and in mammals it was initially observed in the CNS [56–58]. *FoxO6* is expressed in the hippocampus [56–58], is negatively regulated by insulin/IGF signaling via the PI3K-Akt pathway, and is phosphorylated in contrast to other FoxO factors. However, its nuclear localization is not affected by phosphorylation [56, 59]. In this report we demonstrate specific FoxO6 expression patterns in craniofacial structures and show that localized transduction of Hippo signaling controls growth of the anterior craniofacial region.

*FoxO6* was identified in our bioinformatics analysis of transcription factor gene regulatory networks that are active during craniofacial development. FoxO6 expression is specific and spatially restricted to the anterior regions of the brain and face. Cell-based studies showed that FoxO6 regulated components of Hippo signaling. Our subsequent analyses of *FoxO6* mutant mice showed that *FoxO6* loss-of-function led to enhanced head and craniofacial growth. This growth largely affected the anterior-posterior axis and depended on a Hippo pathway that controls the sizes of the brain, face, jaw and incisors during late stages of development. Fox genes have been shown to play different roles in directing facial growth through skeletal and dental tissue components [60, 61]. Thus, the specific expression and function of *FoxO6* in craniofacial tissues in postnatal stages controls growth of the face and subtle changes in *FOXO6* expression controlling Hippo signaling may define facial morphology.

## Results

We have performed extensive gene expression analyses at embryonic and neonate stages during murine craniofacial and dental development. Our independent bioinformatics data from wildtype and several gene knockout mice models has yielded a gene expression network of transcription factors, epigenetic marks, microRNAs and signaling factors (such as Hippo signaling components) that control the different stages of craniofacial and dental development [60, 62–73]. RNA sequencing and array analyses of multiple mice models, validation of gene expression pathways, networks and specific transcription factor targets has identified *FoxO6* as a new gene involved in craniofacial growth. More importantly, our work on Hippo signaling and using the tools to assay for Hippo signaling components identified FoxO6 as a transcriptional activator of *Lats1/2*.

### *FoxO6* is expressed in the brain, craniofacial tissues and somites

**Fig 1:**
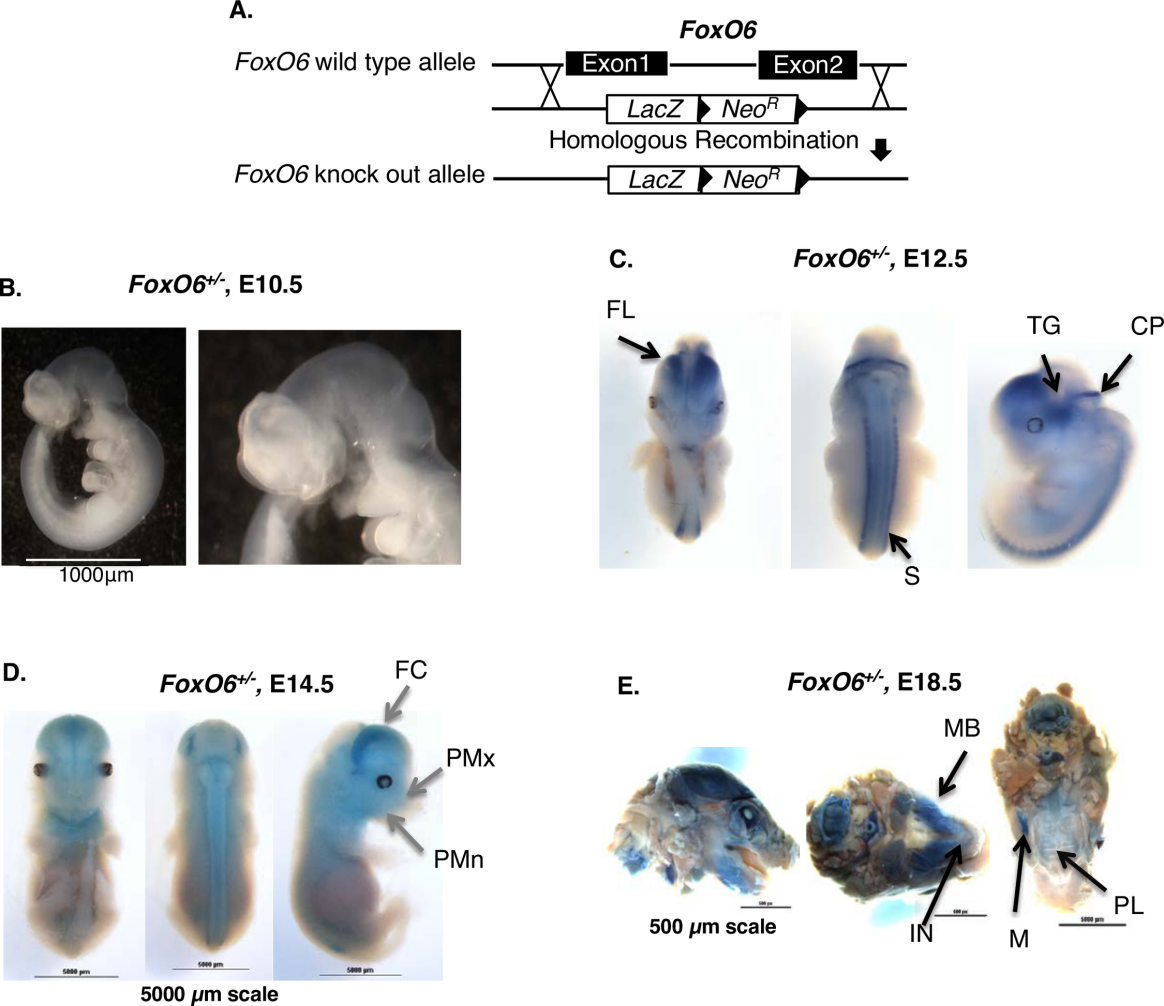
*FoxO6* expression during mouse embryonic development. **A)** Schematic diagram of *FoxO6* knockout strategy. The targeting construct carries a *LacZ* reporter gene and a constitutively expressed neomycin resistance gene (*Neo*) embedded in two loxp sites. The entire *FoxO6* coding region from start codon to stop codon is replaced by *LacZ-Neo* (ES cells obtained from KOMP biorepository). **B-F)** *FoxO6* expression is shown by X-gal staining in *FoxO6*^*+/-*^ mice. **B)** *FoxO6* expression is not detectable at E10.5. **C)** At E12.5, *FoxO6* expression is observed in the brain (frontal lobes, FL; cerebellum primordium, CP; trigeminal ganglion, TG; somites, S. **D)** At E14.5, *FoxO6* expression is in the brain, craniofacial regions, also in the posterior maxilla (PMx) and posterior mandible (PMn). **E)** At E18.5, *FoxO6* has strong expression in the craniofacial region, such as mandibular bone(MB), incisor (IN), molar (M) and palate (PL).

Multiple bioinformatics analyses from different stages of murine craniofacial development comparing WT embryonic and neonate mice to 8 different mouse gene knockout mice identified *FoxO6* as a new gene in craniofacial development. We have identified several new transcription factors involved in craniofacial and dental development based on these data. We generated *FoxO6-lacZ* knock-in mice (*FoxO6*^*+/-*^) from KOMP *FoxO6* gene targeted ES cells (Fig 1A) and performed X-gal staining on staged embryos (Fig 1B–1E). Analysis of whole-mount embryos revealed that at E10.5, FoxO6 was not expressed at detectable levels (Fig 1B); at E12.5 it was expressed within the brain (e.g. frontal lobes, cerebellum primordium and trigeminal ganglion), somites and craniofacial region (Fig 1C); at E14.5, expression was present in the brain and somites, as well as in the posterior regions of the maxilla and mandible (Fig 1D); at E18.5, expression was increased in the craniofacial region (including the maxilla, mandible, incisor, molar and palate) (Fig 1E).

### *FoxO6*^*-/-*^ neonate mouse heads have delayed ossification and a flat skull, while adult heads are expanded anteriorly

**Fig 2:**
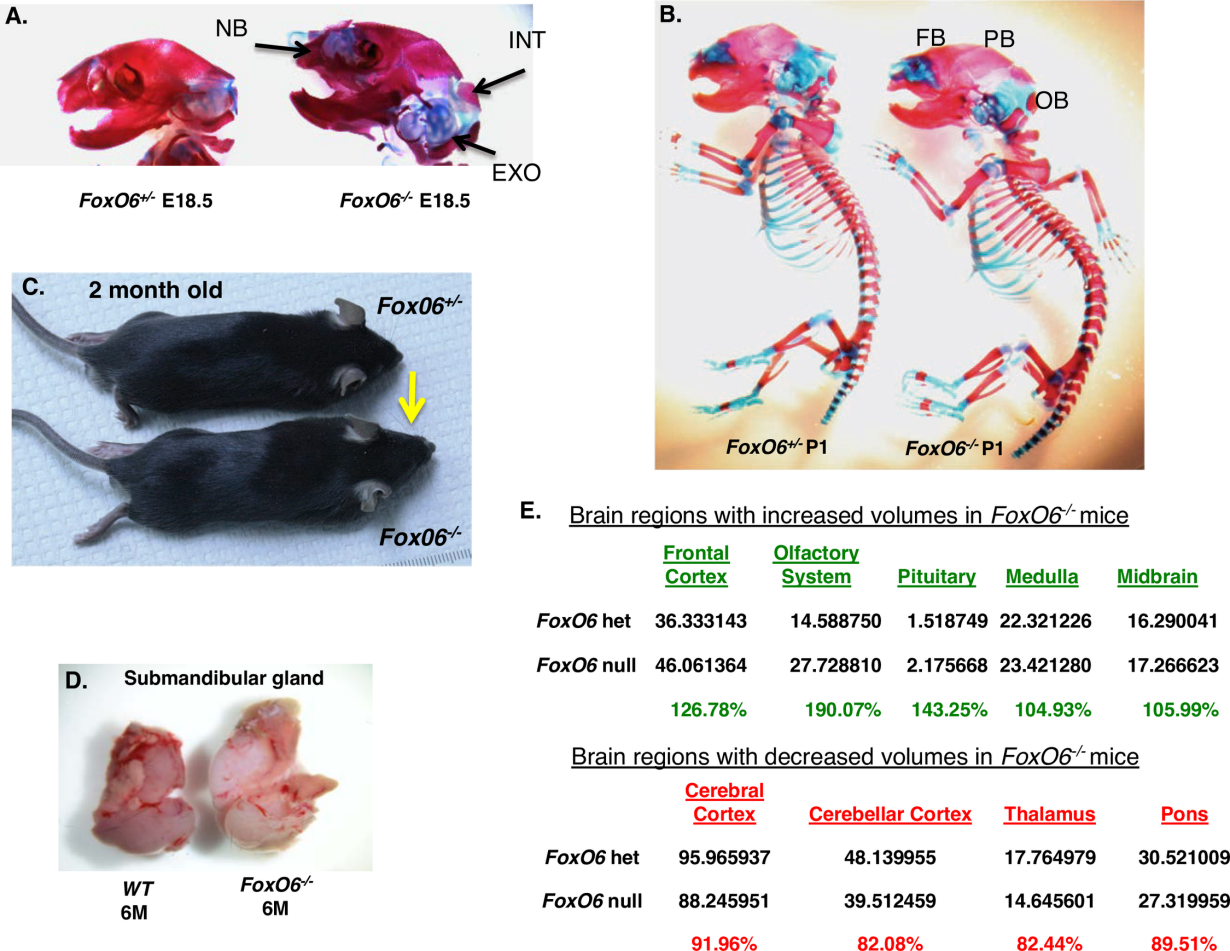
The heads of *FoxO6*^*-/-*^ mice are larger than those of wild type mice. **A,B)** Alcian Blue/Alizarin Red staining of cartilage and bone skeletal preparations of *FoxO6*^*+/-*^ mice and *FoxO6*^*-/-*^ littermates. **A)** E18.5 head skeletal preparations show a larger skull and head in *FoxO6*^*-/-*^ embryos. Moreover, a reduced ossification (red stain) of the interparietal bone (INT), exoccipital bone (EXO) and nasal bone (NB) were observed in E18.5 *FoxO6*^*-/-*^ embryos compared to their heterozygous littermates. **B)** Skeletal preparations of P1 *FoxO6*^*+/-*^ mice (left) and *FoxO6*^*-/-*^ littermates (right) show that *FoxO6*^*-/-*^ mice have an expanded head compared with control mice. The frontal bone (FB), parietal bone (PB) and occipital bone (OB) are slightly larger in *FoxO6*^*-/-*^ mice. **C)** Size of head in 2 month-old *FoxO6*^-/-^ mice compared to WT mice (N>5). The anterior region of the craniofacial complex continues to grow in the *FoxO6*^*-/-*^ mice after weaning, until 2–3 months old (see yellow arrow), while the body size is normal. **D)** The submandibular gland was increased after 6 months in *FoxO6*^*-/-*^ mice compared to WT. **E)** Results of brain MRI scans of 2 month-old control (*FoxO6*^*+/-*^) and *FoxO6*^*-/-*^ mice, comparing the sizes of the different compartments of the brains of *FoxO6*^*+/-*^ and *FoxO6*^*-/-*^ mice. The percent increase in the brain regions are shown on top and the percent decrease on the bottom.

To determine if formation of the cranial bones was affected during late stages of embryonic development, we stained *FoxO6*^*-/-*^ embryos with Alcian Blue/Alizarin Red. This staining revealed that in E18.5 embryos, ossification (red stain) of the interparietal bone (INT), exoccipital bone (EXO) and nasal bone (NB) appeared delayed in *FoxO6*^*-/-*^ embryos (Fig 2A). At P1, the frontal (FB), parietal (PB) and occipital bones (OB) were slightly larger in the *FoxO6*^*-/-*^ mice, with a flattened dorsal skull (Fig 2B). Taken together, these bone staining data indicate that osteogenesis, and in particular endochondral ossification, was modestly delayed in the *FoxO6*^*-/-*^ neonate mice. At birth, the growth of the *FoxO6*^*-*/-^ mice appear normal compare to *FoxO6*^*+/-*^ mice. However, as growth of the mice continued, a significant growth increase of the anterior region of the mandible, maxilla and skull was observed in the *FoxO6*^*-/-*^ mice shown at 2 months of age (Fig 2C). In contrast, body growth of the *FoxO6*^*-/-*^ mice is normal compared to *FoxO6*^*+/-*^ mice. The submandibular gland was enlarged in the 6-month old *FoxO6*^*-/-*^ mouse (Fig 2D).

### In the *FoxO6*^*-/-*^ mice, growth of various cranial regions are affected differently

Magnetic resonance imaging (MRI) analyses of the *FoxO6*^*-/-*^ mice and subsequent volumetric measurement indicated that specific areas of the brain and craniofacial regions differed with respect to growth effects (Fig 2E). The frontal cortex, olfactory system and pituitary, regions of high *FoxO6* expression, were all clearly larger in the *FoxO6*^*-/-*^ mice, while midbrain and medulla were slightly larger than in their *FoxO6*^*+/-*^ littermates (Fig 2E). In contrast, the cerebral cortex, cerebellar cortex, thalamus and pons were all smaller in *FoxO6*^*-/-*^ mice than in controls (Fig 2E). These measurements are consistent with findings from an earlier study on the consequences of reduced hippocampal function for memory and synaptic function in *FoxO6*^*-/-*^ mice [58]. Because *FoxO6* is expressed mainly in the brain and craniofacial region, it acts as a regulator of head morphology. Furthermore, *FoxO6* is expressed at later embryonic stages (after E10.5) and does not appear to affect cell/tissue-specific patterning.

### Ablating *FoxO6* results in specific structural changes in murine head morphology

**Fig 3:**
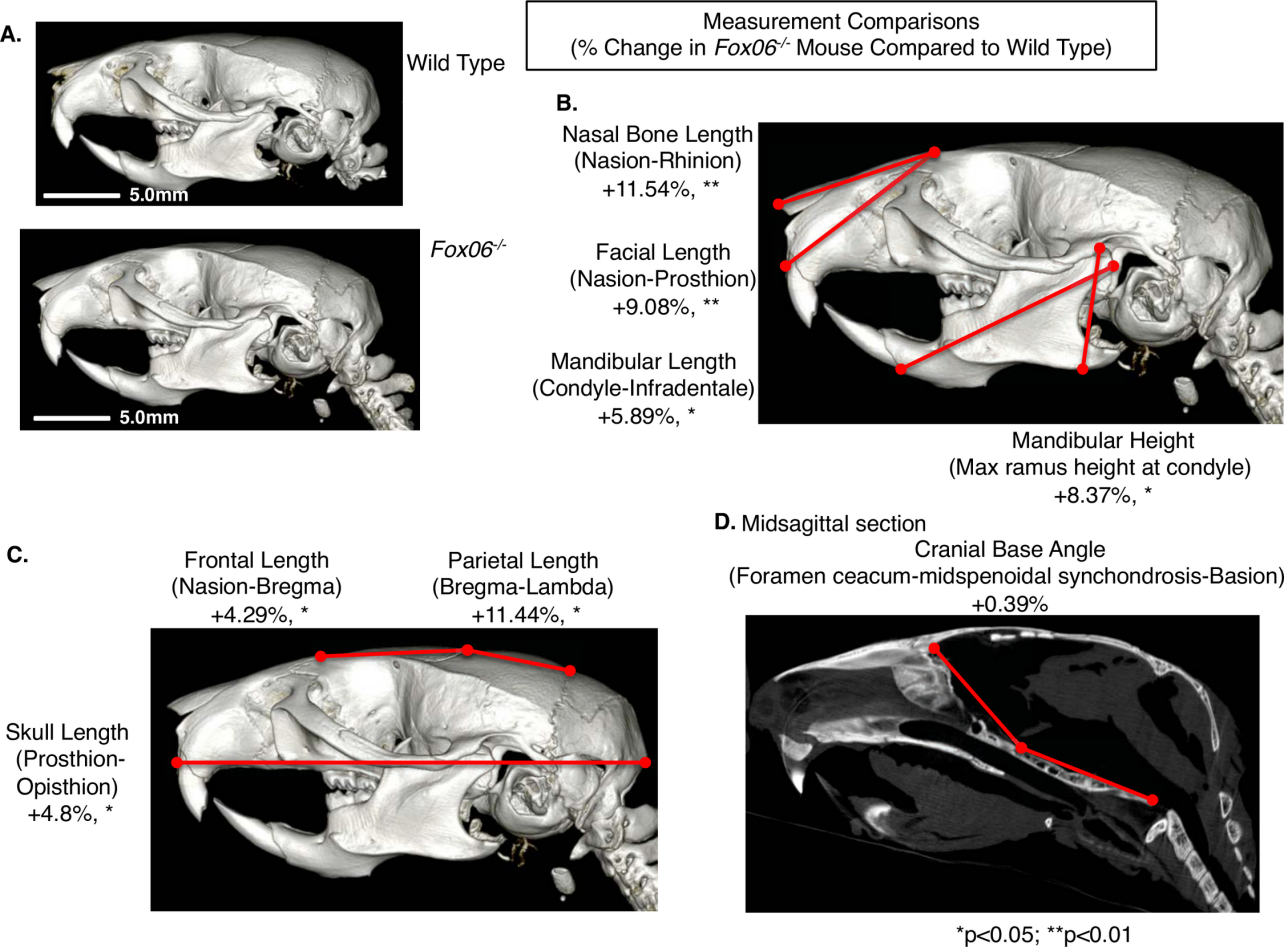
The heads of *FoxO6*^*-/-*^ mice are expanded in the anterior direction. **A-D)** Microcomputed tomography (μCT) analysis of 2 month *FoxO6*^*-/-*^ and WT mice heads. **A)** μCT of wild type and *FoxO6*^*-/-*^ heads used for morphometric measurements. **B-D)** Morphometric measurements of bone length and percent (%) change between *FoxO6*^*-/-*^ and WT mice (N = 3 for each). **B)** Measurements of the nasal length, facial length, mandibular length and mandibular height are all increased in the *FoxO6*^*-/-*^ mouse head, N = 3. **C)** The frontal length, parietal length and skull length were also increased in the *FoxO6*^*-/-*^mouse head, N = 3. **D)** Midsagittal section measurements showed a slight increase in the cranial base angle, N = 3.

Microcomputed tomography (μCT) analysis of whole heads of 2 month-old *FoxO6*^*-/-*^ and WT mice identified specific changes in growth patterns in the context of loss of *FoxO6* function (Fig 3). Lengths of the nasal and facial bones were approximately 10% greater in the mutant animals, N = 3 (Fig 3B). Mandibular length and height were larger by 5.89% and 8.37% respectively, N = 3 (Fig 3B). The lengths of the frontal bone, parietal bone and skull overall were ~4.8% greater (N = 3) (Fig 3C). A midsagittal section measuring the cranial base angle showed that it was similar in the *FoxO6*^*-/-*^ mice compared to WT, N = 3 (Fig 3D). Overall the base length of the cranium was ~7% larger, with increased lengths in the palate, cranial and lower incisors (Fig 3B). Quantitation of other specific craniofacial growth measurements show growth of the palate and lower incisor, however the molars are not significantly increased in length or breadth (S1 Table). These increases in anterior growth became apparent immediately after birth and continued through 2 months of age. These data suggest that *FoxO6* functions after neural-crest migration has taken place and once tissue identity, including that of epithelial tissues, has been specified.

### The position of the lower incisor in the jaw correlates with increased anterior growth of the mandible

**Fig 4:**
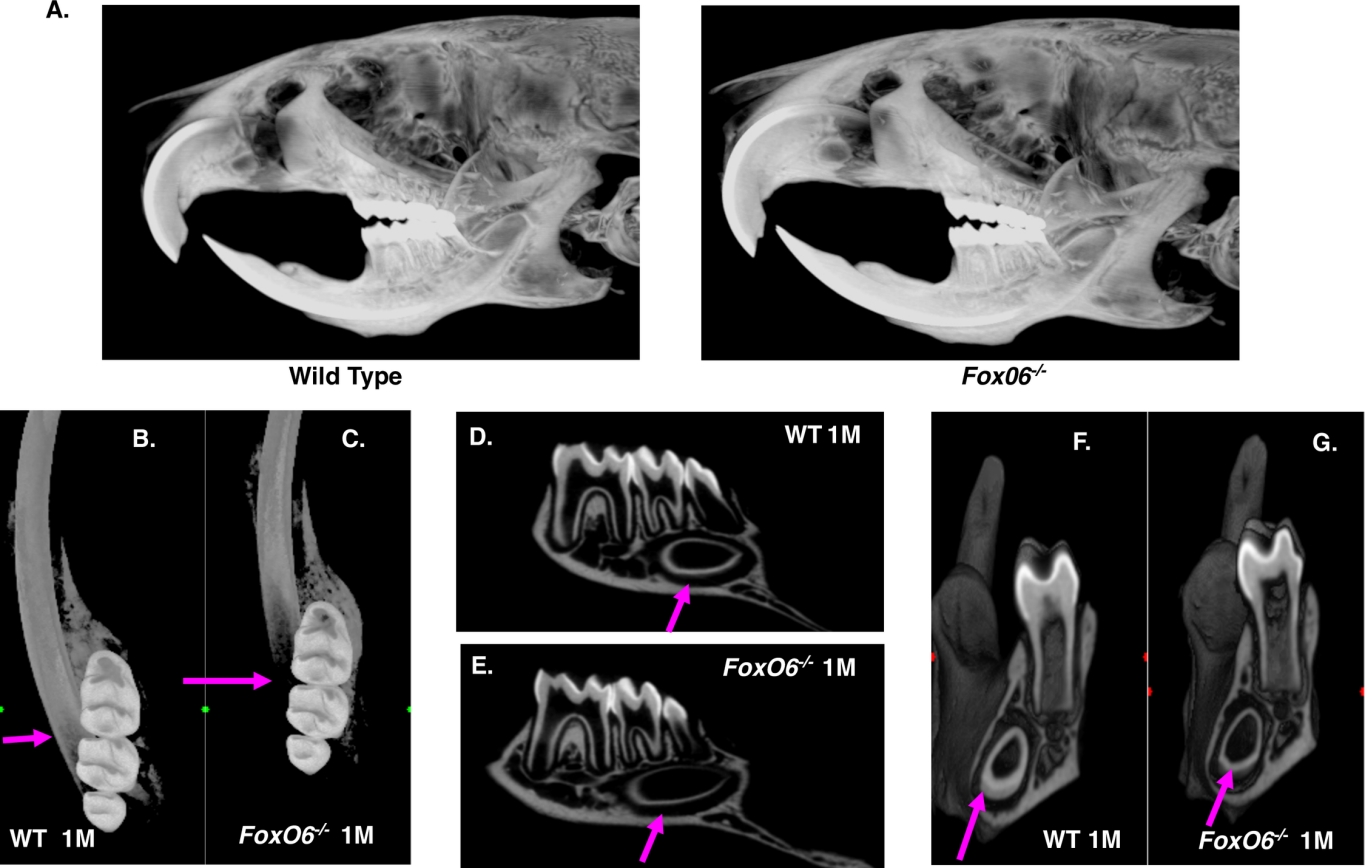
*FoxO6*^*-/-*^ mouse incisors are positioned anteriorly and distal to the molars compared to WT mice. **A-G)** Microcomputed tomography (μCT) analysis of 1 month-old *FoxO6*^*-/-*^ and WT mice heads, mandibles, lower incisors and molars. **A)** μCT maximum projection images of *FoxO6*^*-/-*^ and WT heads used for analyses. **B, C)** Mineralization of the *FoxO6*^*-/-*^ lower incisor resulting in detectable mineral density of enamel is shifted more anteriorly and distally relative to the first molars, the commonly used anatomical landmark for stages of enamel mineralization, compared to the WT incisor (pink arrows). **D, E)** Hemimandible seen in parasagittal plane through the molars showing less mineralization of enamel and dentine, indicative of an anteriorly extended delay of mineralization in the *FoxO6*^*-/-*^ lower incisor at this position (pink arrows). **F, G)** Image of mandibles anterior to the plane through the distal root of the first molar in coronal plane showing that the *FoxO6*^*-/-*^ lower incisor at this posterior position has thinner dentin and decreased enamel formation compared to the WT incisor (pink arrows).

MicroCT analyses of the lower incisor showed a 4.21% increase in lower incisor length (Fig 4A, measurements not shown, N = 3). Although the molars of 1 month-old *FoxO6*^*-/-*^ mice were normal, the lower incisor mineralization was delayed and displaced in the anterior direction due to the overall increase in mandible length (Fig 4B and 4C). Moreover, lower incisor mineralization was not detected in the area of the mesial apex of the second molar, where in control mice enamel mineralization is clearly seen (Fig 4B and 4C, see arrows). In the posterior region, enamel is not detected near the mesial bifurcate root of the first molar in the *FoxO6*^*-/-*^ mice and dentin is thinner and less mineralized (Fig 4D, 4E, 4F, and 4G). Thus, the lower incisor is longer in the *FoxO6*^*-/-*^ mice, and the zone of incisor formation and appositional enamel growth is extended and positioned further anterior relative to the molars, reflecting the increase in anterior growth of the jaw.

### *FoxO6* regulates odontogenesis

**Fig 5:**
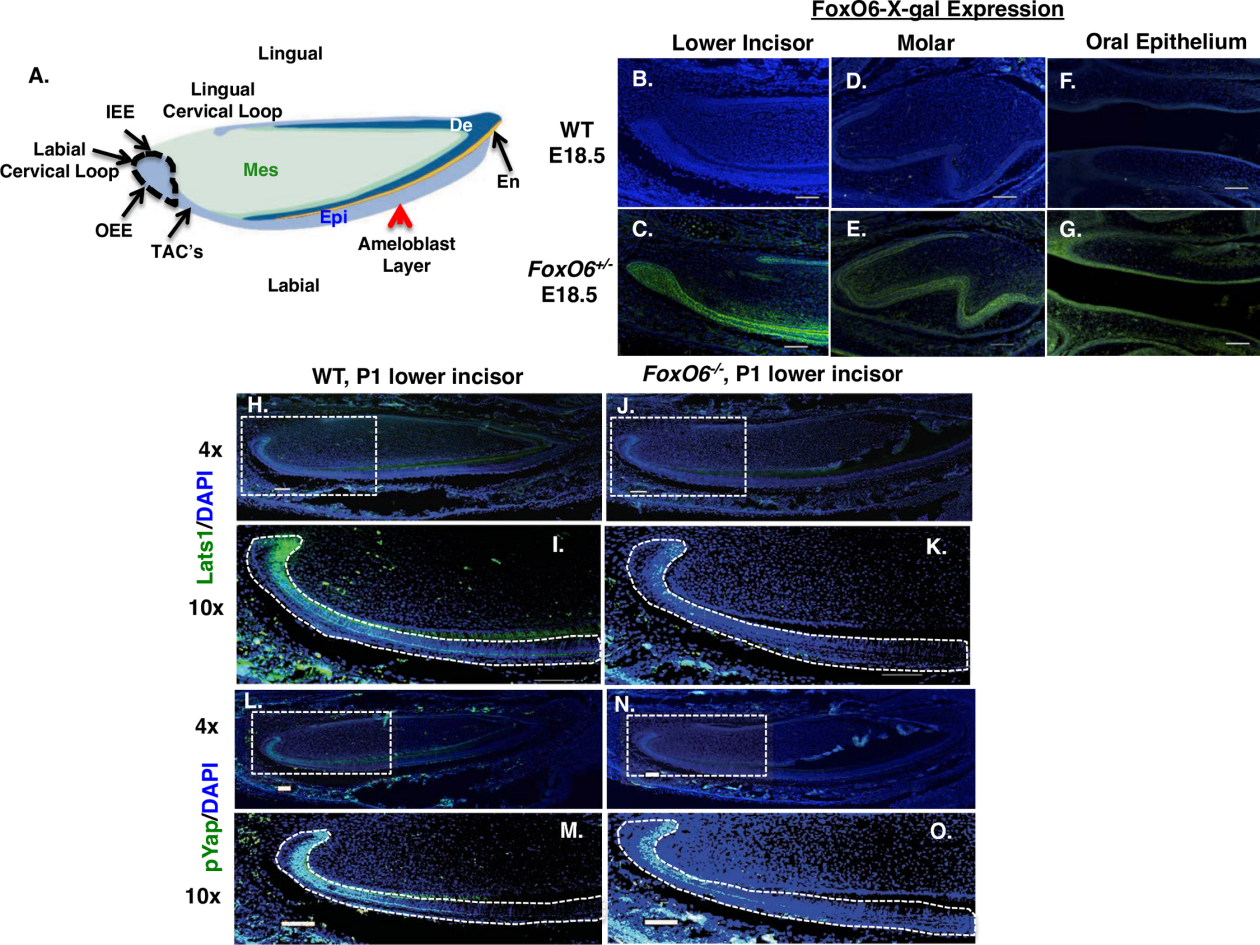
FoxO6 regulates *Lats1* and pYAP expression. **A)** Schematic illustration of the mouse lower incisor, showing the labial cervical loop (LaCL, stem-cell niche), inner enamel epithelium (IEE), outer enamel epithelium (OEE), and ameloblast layer derived from the LaCl cells. **B-G)** *FoxO6* expression in the E18.5 lower incisor (B, C), molar (D, E) and oral epithelium (F, G). *FoxO6* expression indicated by immunostaining using an X-gal antibody (green) of WT (B, D, F) and FoxO6^*+/-*^ (C, E, G) mice (only *FoxO6*^*+/-*^ mice express lacZ). Scale bars represent 100μm. **H-K)** *Lats* expression in *FoxO6*^*-/-*^ vs WT mice. A series of sagittal sections of lower incisors from P1 animals were examined by immunofluorescence staining for *Lats1* protein, using an Alexa-488 labeled antibody. **I, K)** Magnified views of the boxed regions in (H) and (J), respectively. The LaCL, dental epithelium and ameloblast layer is outlined. **L-O**) Expression of pYap. Less Yap is phosphorylated (activated) in incisors of *FoxO6*^*-/-*^ vs WT mice. **M, O)** Magnified views of the boxed regions in (L) and (N), respectively. In all sections, staining was used to identify nuclei. Scale bars represents 100μm.

**Fig 6:**
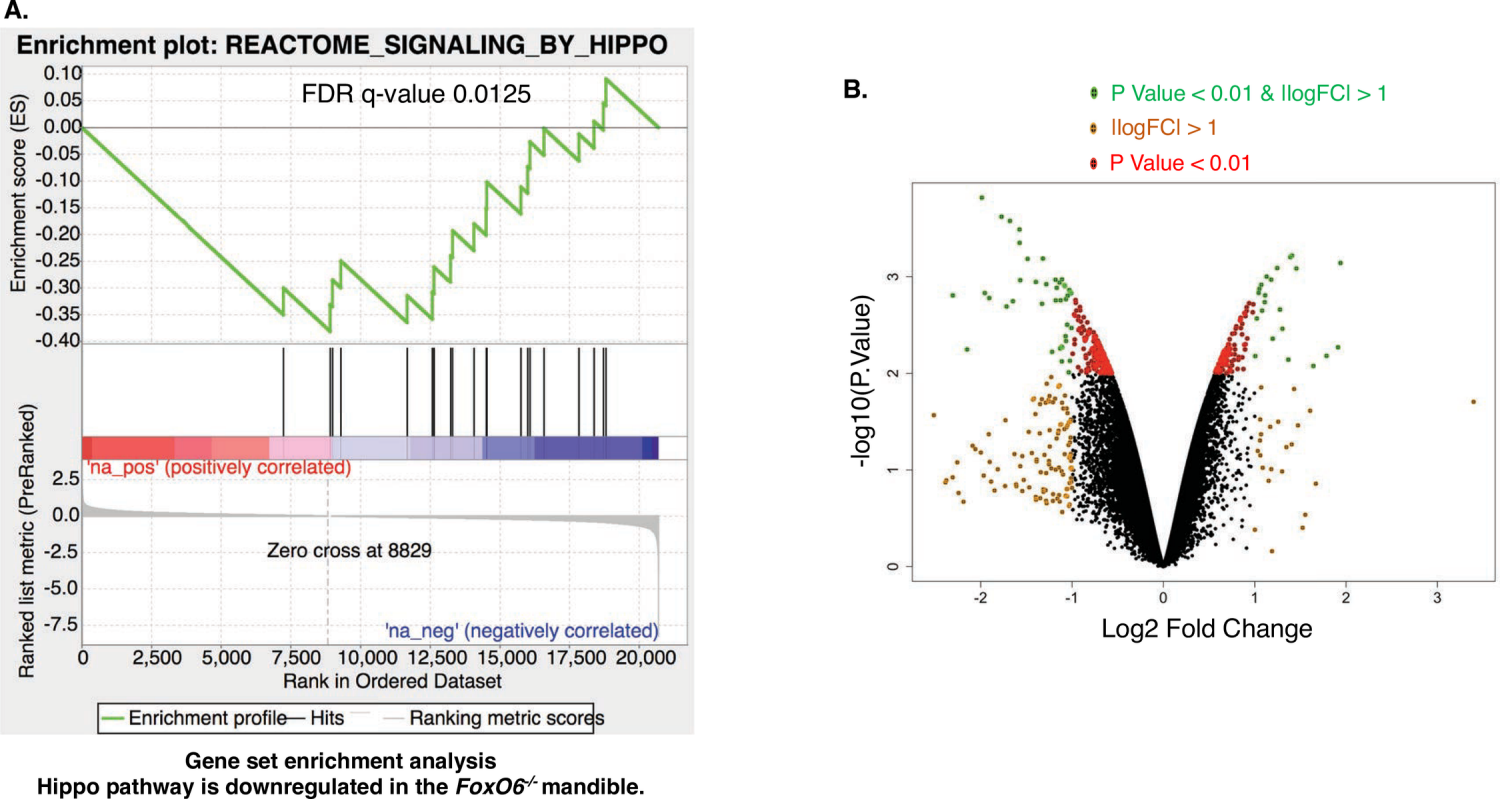
GSEA plot showing the down regulation of the Hippo pathway in *FoxO6*^*-/-*^ mandibular tissue. **A)** All genes are ranked based on fold change between *FoxO6* null and wild type mandible and maxilla tissue, with most up regulated genes (red color) in the left and most down regulated genes (blue color) in the right. Each black line represents a gene in the Hippo pathway. As shown in this figure, Hippo pathway genes tends to located in the right (down regulated) region. **B)** A volcano plot showing multiple genes regulated by FoxO6, both increased and decreased expression (4 biological replicates of WT and *FoxO6* mutant embryos were combined to yield the bioinformatics data).

We used the incisor as one model to determine the role of *FoxO6* in craniofacial development as incisor development is linked with craniofacial development. The mouse incisor is a useful model, since it grows continuously throughout the life-time of the animal, relying on a stem-cell niche in the labial cervical loop (LaCL). Initially, during development, the teeth grow with the mandible and maxilla. However, as the incisors are worn down, the tooth structure is regenerated by stem-cell proliferation and differentiation in the posterior region of the tooth. Fig 5A depicts the rodent lower incisor, including the structures that give rise to the enamel (En)-forming ameloblast, i.e. the: labial cervical loop (LaCL, outlined by dashed line), outer enamel epithelium (OEE), and inner enamel epithelium (IEE). The dental mesenchyme (Mes) also contains stem cells; these give rise to odontoblasts, which produce dentin (De). We analyzed gene expression and epithelial-cell proliferation and differentiation in the lower incisors of *FoxO6*^*-/-*^ mice and WT littermates at E16.5, E18.5 and P0 to study defects in incisor morphogenesis. Bioinformatics analyses of the lower incisors, mandible and maxilla regions demonstrated genes involved in Hippo signaling were regulated by *FoxO6*. Gene expression data has been deposited (GSE117013) in the NIH GEO repository. A heat map of selected genes including Lats1 and Last2 are shown decreased in the *FoxO6*^*-/-*^ mandibles (S1A Fig). A gene ontology map of biological processes shows that FoxO6 regulates transcription (S1B Fig). A gene set enrichment analysis (GSEA plot) shows that the Hippo pathway is down regulated in the *FoxO6*^*-/-*^ mandible (Fig 6). A volcano plot of the RNA-Seq data reveals several genes regulated by FoxO6, including the Hippo pathway (Fig 6). *Lats1* is a major component of the Hippo pathway and it was significantly decreased (P< 0.01) in the *FoxO6* null mouse mandible tissue (Figs 6 and S1; RNA from 4 biological samples combined for bioinformatics analyses).

We used X-gal antibody to probe FoxO6 expression in E18.5 sagittal sections of the *FoxO6*^*+/-*^ mouse head. X-gal was highly expressed in the dental epithelium (both incisor and molar) (Fig 5C and 5E) oral epithelium and craniofacial mesenchyme (Fig 5G). This is consistent with a role for *FoxO6* in both odontogenesis and development of the mandible and maxilla. Notably, FoxO6 was detected in transit amplifying cells (TACs) [74] of the dental mesenchyme as well (Fig 5C and 5E). We further performed immunostaining using a FoxO6 antibody to probe for FoxO6 expression and compare to the X-gal staining. FoxO6 was specifically expressed in the oral epithelium (OE), dental lamina (DL) and lower incisor dental epithelium (S2A–S2D Fig). Low level of expression was observed in the dental mesenchyme and no FoxO6 expression was seen in the *FoxO6*^*-/-*^ embryos. RNA extracted from WT and *FoxO6*^*-/-*^ mandibles shows no *FoxO6* transcripts were present in the *FoxO6*^*-/-*^ embryos (S2E Fig).

To demonstrate a role for FoxO6 in the direct regulation of Hippo signaling we used a Hippo reporter construct (HOP) that contains several TEAD binding sites to activate luciferase expression in CHO cells. A Hippo reporter with mutations in the TEAD binding sites (HIP) acted as a control reporter. Cotransfection of FoxO6 with the HOP reporter resulted in decreased HOP activation, while the HIP reporter was not affected (S2F Fig). In contrast knockdown of FoxO6 expression (shFoxO6) resulted in an increase in HOP reporter activity (S2F Fig). As controls the constitutively active YAP 5SA construct was cotransfected with HOP and HIP and showed that YAP 5SA activated the HOP reporter as expected (S2F Fig). Cotransfection of WT YAP had only a modest effect on HOP activity. These results demonstrate that FoxO6 has a direct effect on Hippo signaling through regulating YAP activation of the HOP reporter.

Given that our analysis of gene expression arrays had shown Hippo signaling in *FoxO6*^*-/-*^ mice to be defective, and that our bioinformatics analyses demonstrated that Lats1, Yap and pYap were expressed during incisor development (S1 Fig), we sought to confirm the dental effects of defective Hippo signaling in *FoxO6*^*-/-*^ mice. Immunofluorescence assays demonstrated that Lats1 is normally expressed in the lower incisor (LI) of the P1 mouse, and that Yap is phosphorylated at this time. In WT, sagittal sections of the lower incisor, shows Lats1 expression is predominantly localized to the LaCL region and to some extent in the dental mesenchyme (Fig 5H and 5I). As expected, in the *FoxO6*^*-/-*^ P1 mouse this expression was lower (Fig 5J and 5K). Examination of pYap staining in sections of WT LI’s revealed that Yap was activated in the LaCL, differentiating ameloblasts and mesenchymal cells (Fig 5L and 5M). In the *FoxO6*^*-/-*^ P1 mice, this expression was lower (Fig 5N and 5O). These experiments suggest that FoxO6 directly activates Lats1 and Yap *in vivo* to regulate Hippo signaling during incisor development.

**Fig 7:**
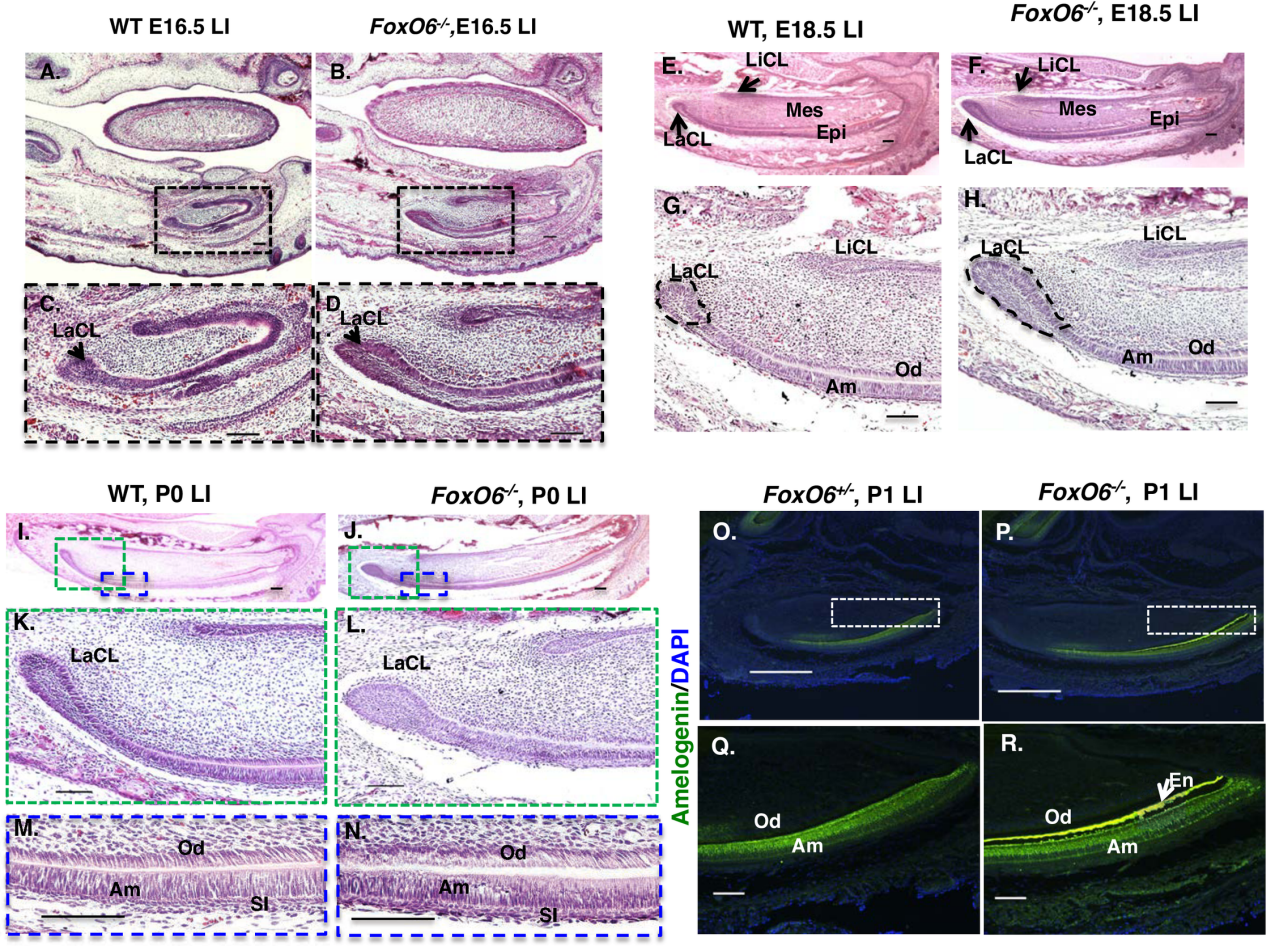
In *FoxO6*^*-/-*^ mice, the incisors are enlarged, the LaCL is expanded and the polarity of the dental epithelium is abnormal. Sections of incisors from E16.5, E18.5, and P0 WT and *FoxO6*^*-/-*^ mice stained with Hematoxylin and Eosin. **A-B**) E16.5 mandibles, with lower incisor (LI) framed by box to highlight differences in incisor size. **C-D**) Higher magnification of the boxed region of the lower incisors in A and B, showing that they are enlarged and that they have larger labial cervical loops (LaCLs) in *FoxO6*^-/-^ embryos. **E-F)** In E18.5 mandibles, a longer lower incisor is still observed in *FoxO6*^*-/-*^ embryos. **G-H)** Higher magnification of E and F (LaCL is outlined in black). **I-J)** P0 mandible. The lower incisor and LaCL are much larger in the *FoxO6*^*-/-*^ mice. **K-L)** Higher magnification of the red box of I and J, revealing that the LaCL is larger in *FoxO6*^*-/-*^ mice. **M-N)** Higher magnification of the ameloblast and odontoblast cell layers (blue boxed region in I, and J) show that the cells are not well polarized in *FoxO6*^*-/-*^ mice compared to WT mice. **O,P)** Amelogenin staining in the lower incisor (LI) of *FoxO6*^*+/-*^ P1 mice compared to *FoxO6*^*-/-*^ littermates. **Q,R)** Higher magnification of the boxed area in O and P showing amelogenin in the ameloblast layer. Increased enamel (En) formation is observed in the *FoxO6*^*-/-*^ mice. Scale bar represents 100μm. Abbreviations: Epi, dental epithelia; Mes, dental mesenchyme; LaCL, labial cervical loop. LiCL, lingual cervical loop; Am, Ameloblast; Od; Odontoblast; SI; stratum intermedium.

Consistent with a role for *FoxO6* in regulating Hippo signaling, we observed an increase in the size of the lower incisors of E16.5 and E18.5 (Fig 7A–7H), as well as of the LaCL, in *FoxO6*^*-/-*^ embryos (LaCl is outlined in G and H). These differences persisted in P0 *FoxO6*^*-/-*^ mice (Fig 7I–7L), at which point structural defects in ameloblasts (dental epithelium, Am) and odontoblasts (dental mesenchyme, Od) were also noted in the *FoxO6*^*-/-*^ mice, with neither cell layer as uniform as in WT mice (Fig 7M and 7N). We asked if the ameloblasts in the *FoxO6*^*-/-*^ incisors indeed express amelogenin, the most abundant structural enamel matrix protein that is required for the formation of enamel and that marks the differentiation of this cell type. Immunofluorescence analysis using an amelogenin antibody revealed greater overall expression of this protein, due to an increase in length of this structure and a corresponding increase in the number of differentiated ameloblasts (Fig 7O–7R). Thus, whereas the ameloblast layer is less structured in *FoxO6*^*-/-*^ mice, the cells differentiate as ameloblasts. Thus, FoxO6-mediated, Hippo-dependent regulation of incisor size in coordination with craniofacial growth may be due to the control of LaCL stem-cell proliferation.

### *FoxO6* modulates cell proliferation

**Fig 8:**
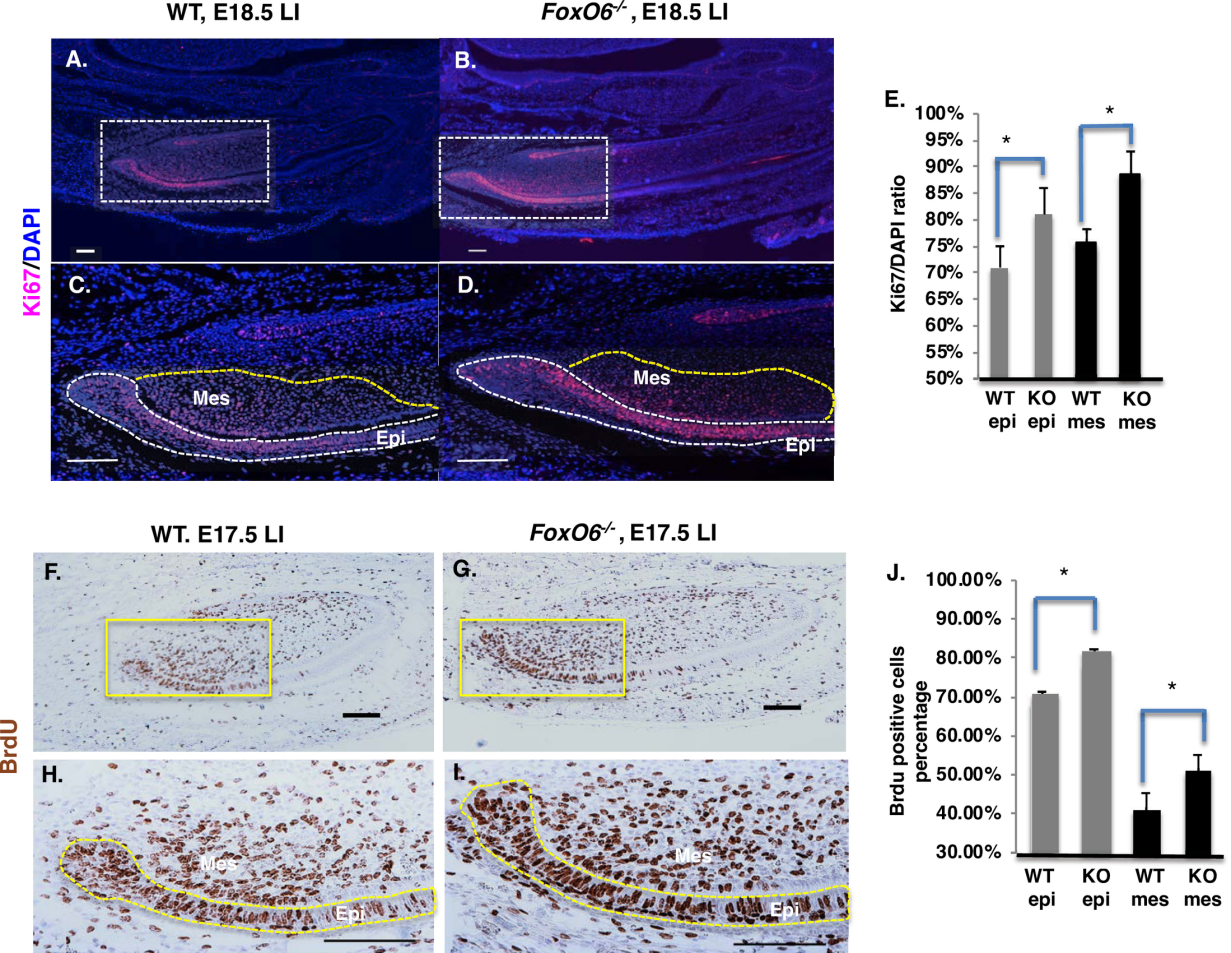
In *FoxO6*^*-/-*^ mice, the incisors exhibit increased cell proliferation. **A-B)** Cell proliferation in lower incisors from WT or *FoxO6*^*-/-*^ E18.5 embryos, as assessed by staining with Ki67 and DAPI. **C-D)** Higher magnification of the LaCL region from WT or *FoxO6*^*-/-*^ embryos in A and B. The Ki67 positive cells are located mainly in the Transient Amplifying (TA) zone of the IEE and adjacent mesenchymal tissue. **E)** Quantitation of the Ki67-positive cells in the sections of lower incisors. The number of Ki67-positive cells in *FoxO6*^*-/-*^ epithelial and mesenchymal tissue is increased compared to WT embryos. **F-G)** Cell proliferation in E17.5 WT and *FoxO6*^*-/-*^ embryos, as assessed by BrdU injection (2 hours prior to sacrifice). **H-I)** Higher magnification of posterior or proximal lower region of incisor in WT and FoxO6^*-/-*^ embryos in F and G, demonstrating increased epithelial and mesenchymal cell proliferation compared to the WT. **J)** Quantitation of the BrdU-positive cells in sections of lower incisors. Scale bar represents 100μm. Epi, epithelium; Mes, mesenchyme.

Hippo signaling is known to regulate organ size by modulating cell proliferation [8, 75]. To determine if the observed increase in incisor length in of *FoxO6*^*-/-*^ mice was due to an increase in cell proliferation, E18.5 *FoxO6*^*-/-*^ and WT embryos were sectioned and proliferation was assessed using the Ki67 antibody. This analysis revealed an increase in Ki67-positive epithelial cells in mutant vs. WT incisors (Fig 8A and 8B), specifically within the LaCL (Fig 8C and 8D). Quantitative analysis revealed that this increase applied to the epithelium (IEE) and mesenchyme (Fig 8E), indicating that dental-cell proliferation is up-regulated in *FoxO6*^*-/-*^ incisors. BrdU labeling in E17.5 *FoxO6*^*-/-*^ incisors confirmed this increase in cell proliferation (Fig 8F–8I). Quantitation of the BrdU-positive cells demonstrated that the proliferation of both epithelial and mesenchyme cells was increased (Fig 8J). BrdU labeling was subsequently performed in P7 mice to show increase cell proliferation at later stages. Analyses of the lower incisor shows an increase in cell proliferation of the transit amplifying cells of the dental epithelium in *FoxO6*^*-/-*^ mice compared to WT mice (S3A and S3B Fig); white line denotes transit amplifying cells of the lower incisor). Quantitation of cell proliferation demonstrates a 20% increase in epithelial cell proliferation (S3C Fig). To independently show that FoxO6 regulates cell proliferation, CHO cells were transfected with either FoxO6, a short hairpin FoxO6 inhibitor (pshFoxO6) or empty vector DNA plasmids. The over expression of FoxO6 inhibited cell proliferation while inhibition of FoxO6 increased cell proliferation (S3D Fig).

The activation of Hippo signaling inhibits cell proliferation, and we have shown that FoxO6 activates Lats1 expression and increases the phosphorylation of YAP. This modification is known to cause Yap to be sequestered in the cytoplasm and to thereby down-regulate the expression of genes required for both cell proliferation and anti-apoptotic activity. Thus, in the absence of *FoxO6* the Hippo pathway is not stimulated, and this leads to specific tissue responses involving cell proliferation.

### *FoxO6* activates *Lats1* to promote Yap phosphorylation

**Fig 9:**
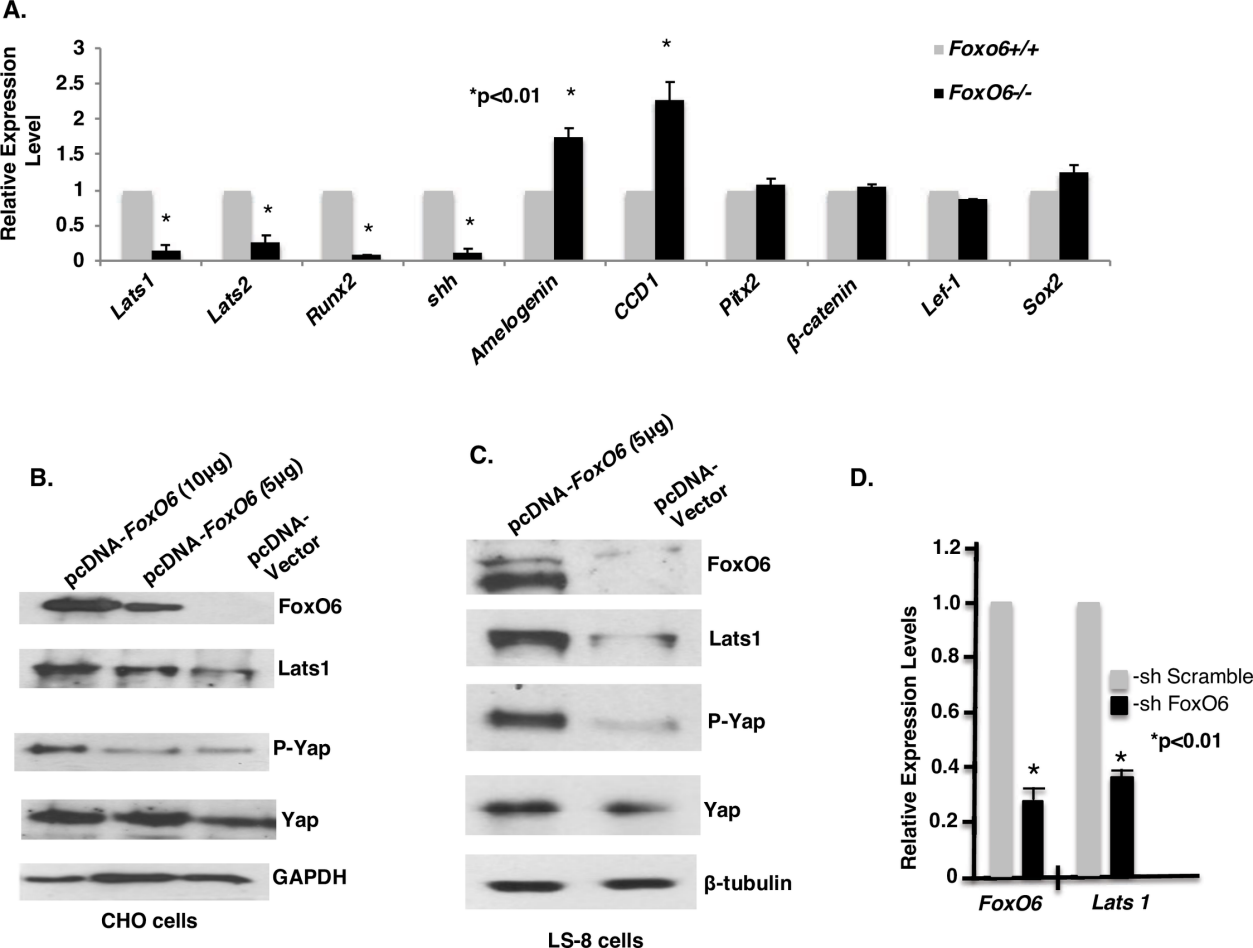
FoxO6 activates *Lats1/2*, *Runx2*, *Shh* and the Hippo signaling pathway. Genes identified as being differentially expressed in *FoxO6*^*-/-*^ and WT mandibles in microarray screen were assessed. **A)** Relative expression of genes in mandible and maxilla tissue of E18.5 *FoxO6*^*-/-*^ and WT (*FoxO6*^*+/+*^) was assessed by real-time PCR and normalized to expression of *β-actin*. Experiments were repeated at least three times each, from multiple biological samples. Error bars indicate S.E. *: p-value<0.01. **B)** Expression and phosphorylation of gene product in CHO cells over-expressing *FoxO6*. CHO cells were transfected with 5 μg empty vector (control), 5 μg pcDNA-*FoxO6*, or 10 μg pcDNA-*FoxO6*. Lysates were collected after 2 days for immunoblotting. **C)** Expression and phosphorylation of gene product in LS-8 (oral epithelial-like) cells over-expressing *FoxO6* over-expression. 5 μg empty vector (control) or 5 μg pcDNA-FoxO6 were used for electroporation. Lysates were collected after 2 days for immunoblotting. **D)** Expression of FoxO6 and Lats1 in ET-16 cells (oral epithelial cells) subjected to *FoxO6* knockdown. 10 μg shScramble DNA or 10 μg *shFoxO6* DNA were transfected and cell lysates were collected after 2 days for RNA extraction and real-time PCR.

In order to find genes downstream of *FoxO6* that mediate craniofacial development, we assessed gene expression using arrays generated from the mandibular and maxillary tissue from E18.5 WT and *FoxO6*^*-/-*^ mouse embryos. Subsequent gene ontology (GO) analysis of biological processes indicated that *FoxO6* plays a role in regulating transcription and development. Expression of *Lats1*, an important component of the Hippo signaling pathway, was ~20-fold lower in *FoxO6*^*-/-*^ mice compared to WT (Figs 6 and S1). To validate these array data and characterize other genes involved in regulating cell proliferation and differentiation, we performed RT-qPCR using the same tissues (Fig 9A). These analyses revealed reduced expression of the following genes: *Lats1* and *Lats2* (~80% and 70% reduced, respectively), *Runx2* (85% reduced), and *Shh* (80% reduced). The observed change in expression in *Runx2* is consistent with the roles of the encoded transcription factor in osteoblast differentiation and skeletal morphogenesis, as well as with the decrease in ossification observed at E18.5 in *FoxO6*^*-/-*^ mice (Fig 2A and 2B). The difference in *Shh* expression is likewise consistent with the role of its product in regulating the size and shape of the face [22]. Genes whose products were expressed at higher levels were *amelogenin* and *cyclin D1*. The former is a marker for the differentiation of ameloblasts and the formation of dental enamel, and the latter is a positive regulator of cell proliferation.

We next used an *in vitro* assay to test whether *FoxO6* regulates Hippo signaling through *Lats1*. To this end, we over-expressed *FoxO6* in CHO and LS-8 cells (oral epithelial-like cells) by transient transfection. This led to an increase in Lats1 expression in both cell lines (Fig 9B and 9C), and the specificity of the effect was supported by the responsiveness to Fox06 dosage in the CHO cells (Fig 9B). Notably, the level of Yap phosphorylation was also higher in both of the transfected cell lines (Fig 9B and 9C) even though overall levels of Yap expression did not differ (Fig 9B and 9C). Subsequent analysis of the effects of *FoxO6* knockdown on *Lats1* and *Lats2* in ET-16 cells (oral epithelial cells in which *FoxO6* is highly expressed) by real-time PCR revealed that a 70% reduction in Fox06 was accompanied by a 60% decrease in *Lats1* (Fig 9D). These data suggest that FoxO6 controls Hippo signaling by regulating *Lats1* expression.

### FoxO6 binds directly to the *Lats1* promoter and activates *Lats1* expression

**Fig 10:**
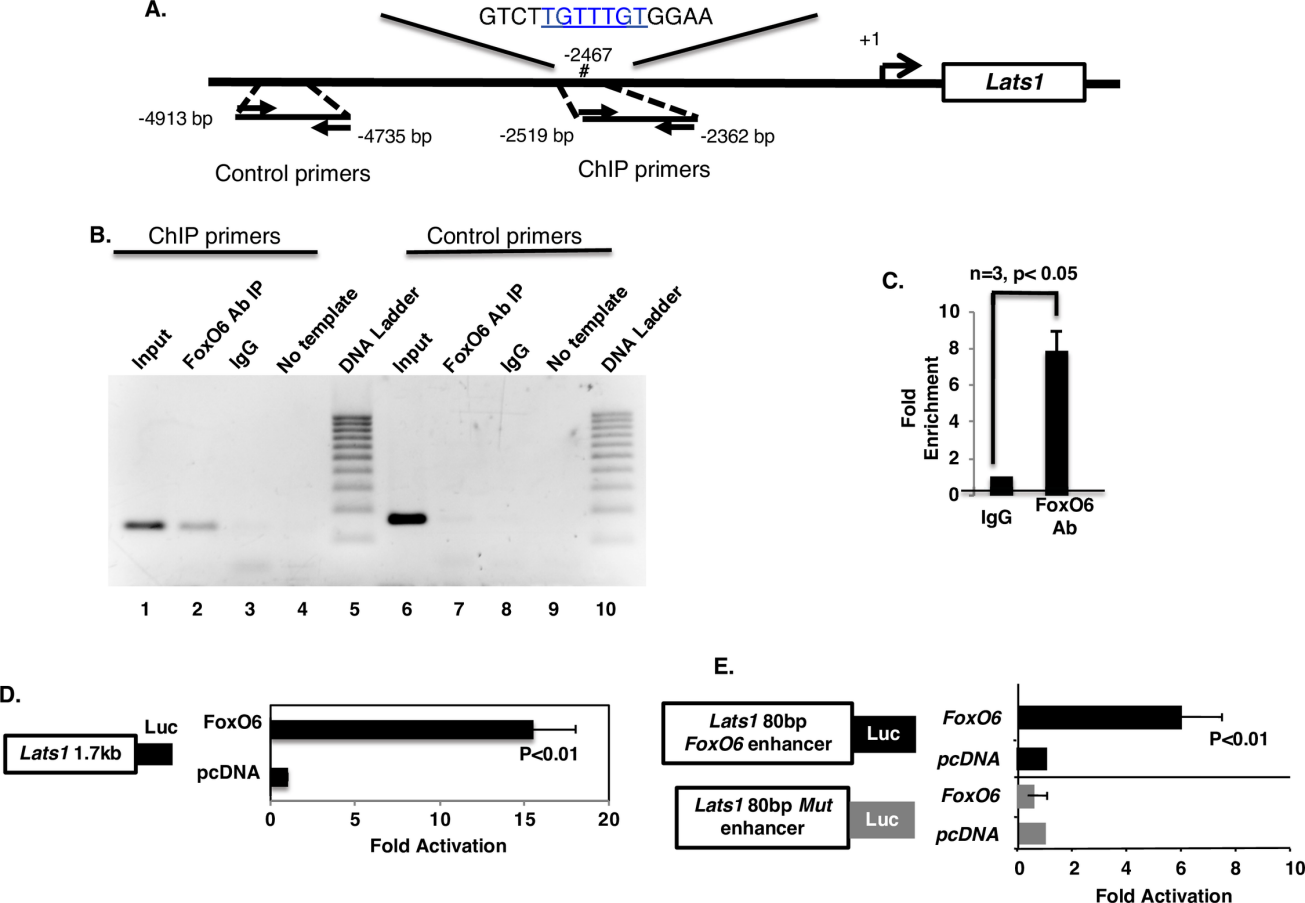
*FoxO6* directly binds to and activates the *Lats1* promoter. **A)** Schematic of the *Lats1* 1.7kb promoter, with the predicted *FoxO6* binding motif (-2467bp). Primers were designed to flank the predicted FoxO6 binding site (-2362 to -2519 bp; ChIP primers) and to an upstream region that lacks a FoxO6 binding site (-4735 to -4913bp; Control primers). **B)** PCR products from ChIP assays involving immunoprecipitation of endogenous *FoxO6* in LS-8 cells. PCR products were resolved in agarose gels. Input chromatin was used as a control to show the primer product; FoxO6 Ab (antibody) immunoprecipitated (IP) the chromatin containing the FoxO6 binding site (lane2); IgG alone did not IP the chromatin and the no template PCR reaction did not produce a band. Control primers to an upstream region of the Lats1 promoter did not detect an IP product with the FoxO6 Ab or the IgG control. **C)** Quantitation of the enrichment of binding by endogenous FoxO6 chromatin compared to IgG immunoprecipitated DNA. **D)** Activation of the Lats1 1.7kb promoter by FoxO6 using a Lats1 luciferase reporter in transfected LS-8 cells compared with empty vector. **E)** Activation of an Lats1 enhancer luciferase reporter whose expression is driven by a duplicated 80 bp DNA fragment derived from the *Lats1* promoter region containing the *FoxO6* binding site. This construct was transfected into LS-8 cells with or without FoxO6 expression plasmid. In parallel, a reporter with a mutated *FoxO6* binding site (Lats1 80bp Mut enhancer) was transfected as control.

Sequence analysis of the *Lats1* 5’flanking region identified a consensus FoxO6 binding site approximately 2,471 bp upstream of the *Lats1* transcription start site (Fig 10A) [58]. Chromatin immunprecipitation (ChIP) assays demonstrated that endogenous FoxO6 binds to this consensus binding site (Fig 10B, lane 2). The chromatin input is shown in lane 1, and the control IgG, which failed to pull down the chromatin, in lane 3 (Fig 10B). Specificity test of the ChIP assay was carried out using primers to an upstream region of the Lats1 promoter that does not contain a FoxO6 binding element. The FoxO6 antibody did not immunoprecipitate chromatin that does not contain a FoxO6 binding site (Fig 10B, lane 7); chromatin input and IgG control are also shown (Fig 10B, lane 6 and 8, respectively). Quantitative PCR demonstrated an 8-fold enrichment of the FoxO6 ChIP product over that of the IgG control (Fig 10C). The *Lats1* promoter was cloned (1.7Kb) upstream of the luciferase gene to measure promoter activity. Co-transfection of the *Lats1* promoter with FoxO6 resulted in 15-fold activation (Fig 10D; comparison is to transfection with empty vector transfection). Mutation of the FoxO6 binding site abolished this activation (Fig 10E). These results indicate that FoxO6 directly activates the *Lats1* promoter.

### *FoxO6* expression is activated by Pitx2, an early regulator of face morphology

**Fig 11:**
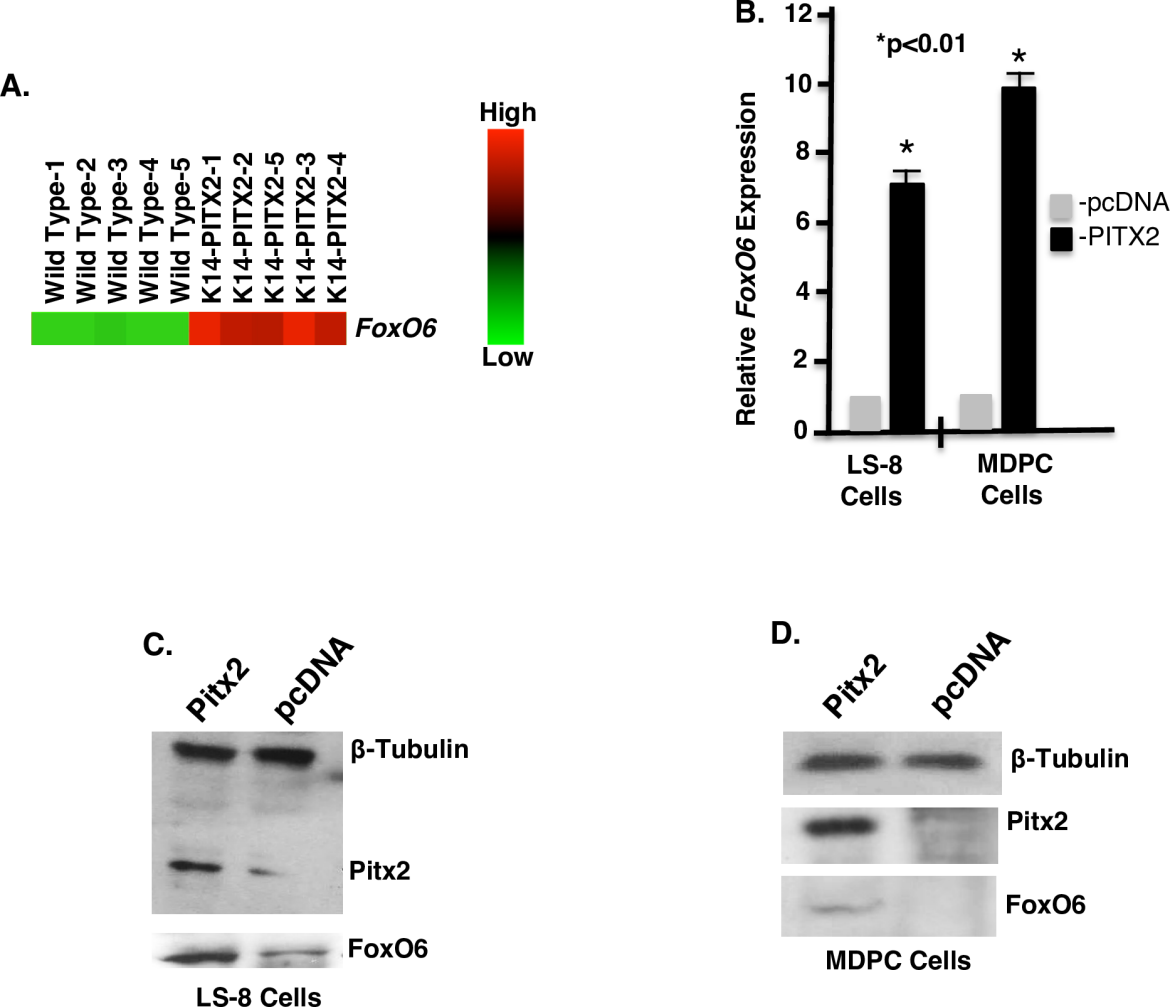
*Pitx2* activates *FoxO6* expression. **A)** Heat map from gene expression microarray showing *FoxO6* is increased in *Krt14-PITX2* overexpression mouse incisor dental epithelium compared to WT. DNA microarray analyses of five different E18.5 mouse dental epithelial tissue samples reveals an increase in *FoxO6* expression in the PITX2 over-expression incisor. **B)** Real-time PCR demonstrates increased endogenous *FoxO6* transcripts in LS-8 cells and MDPC cells that over-express PITX2 compared with empty vector. **C, D)** LS-8 cells (C) and MDPC cells (D) were infected with *Pitx2* or scrambled lentivirus and probed for Pitx2 and *FoxO6* expression by Western Blot. β-Tubulin served as a loading control.

**Fig 12:**
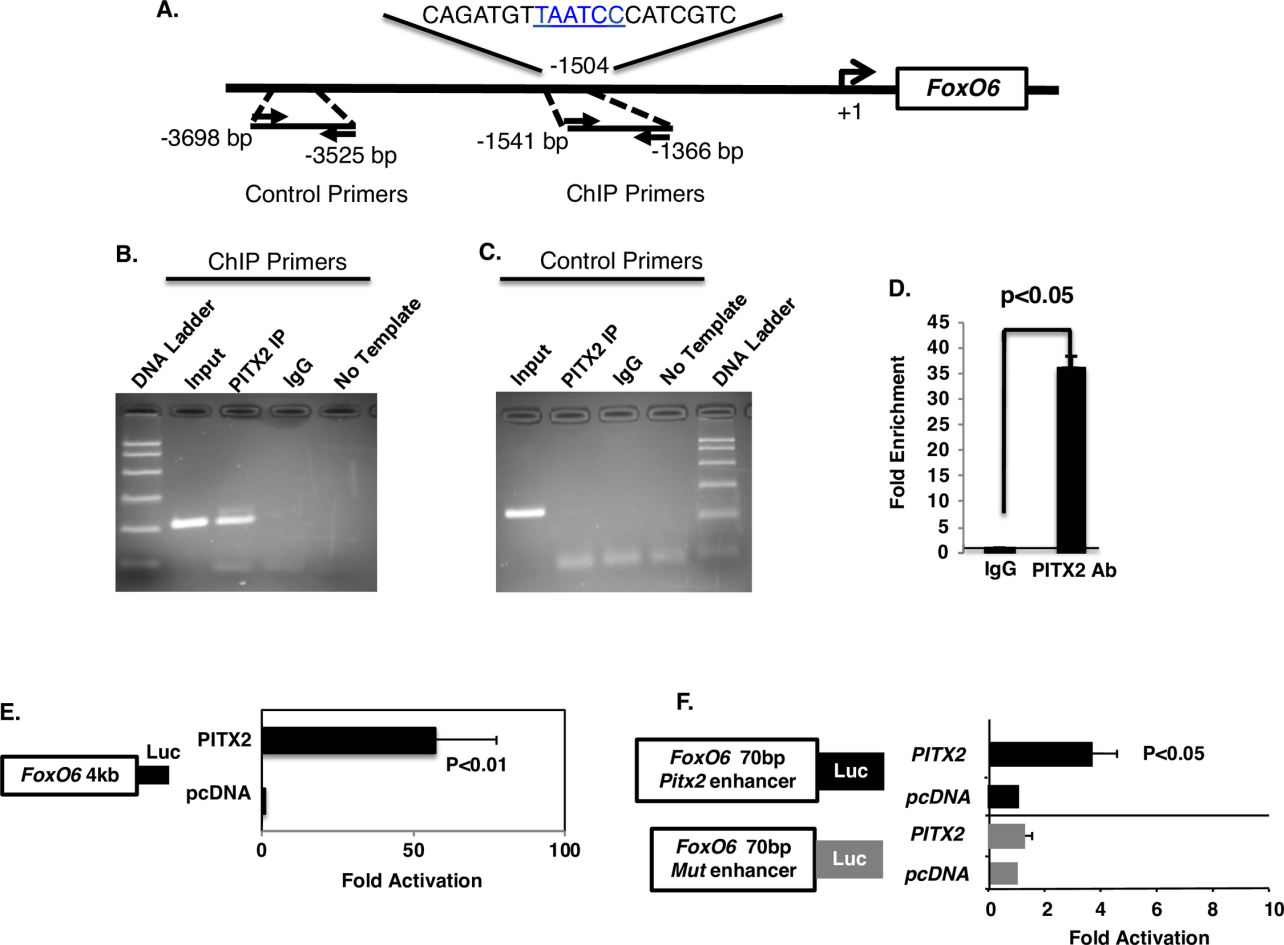
*FoxO6* expression is directly activated by Pitx2. **A)** Schematic of the *FoxO6* 4kb promoter with the Pitx2 binding motif (TAATCC) highlighted by red letters. Primers were designed to flank the predicted Pitx2 binding site (-1366 to -1541bp; ChIP primers) and an upstream region that lacks a Pitx2 binding site (-3525 to -3698bp; Control primers). **B-C)** Endogenous ChIP assay was performed in LS-8 cells. ChIP assay was performed as in Fig 6. **D)** Quantitation of the ChIP assays show a 40-fold enrichment of endogenous Pitx2 binding to the FoxO6 promoter compared to IgG IP. **E)** The *FoxO6* promoter (4 Kb-5’ flanking region) was cloned into a luciferase vector and co-transfected with PITX2 or empty vector in LS-8 cells. Luciferase assays from four independent experiments were analyzed (N = 4). PITX2 activates the FoxO6 promoter at over 50-fold. **F)** A *FoxO6* Pitx2 enhancer element (~70 bp) was cloned into a TK minimal promoter luciferase construct to test for enhancer activity. The *FoxO6* enhancer construct was co-transfected with either PITX2 or empty vector. A similar *FoxO6* enhancer reporter with the mutated Pitx2 binding motif was transfected in parallel as a control. All luciferase activities are normalized to β-galactosidase activity and shown as mean-fold activation compared with the *FoxO6* enhancer plasmid co-transfected with empty CMV expression plasmid (SEM from five independent experiments).

Pitx2 regulates a hierarchical gene regulatory network to control tooth initiation, patterning and growth [62, 63, 76–83]. Human *PITX2* mutations are associated with Axenfeld-Rieger Syndrome (ARS) and these individuals have a flattened mid-face phenotype and tooth agenesis [84, 85]. Pitx2 is also expressed in the oral epithelium, and our bioinformatics analysis of the consequences of the overexpression of PITX2 in mandibular and maxillary tissues show that PITX2 activates *FoxO6* expression (Fig 11A). Consistent with these findings, real-time PCR analysis of PITX2-transfected LS-8 and MDPC cells showed an increase in *FoxO6* transcripts (Fig 11B), and Western blot analyses of PITX2-transfected cells showed a corresponding increase in FoxO6 protein (Fig 11C and 11D). Sequence analyses of the *FoxO6* 5’-flanking region identified a consensus PITX2 binding element 1504 bp upstream of the transcription start site (Fig 12A), and a ChIP assay revealed that endogenous Pitx2 bound to the *FoxO6* chromatin in LS-8 cells (Fig 12B) but not to DNA from an upstream region of the *FoxO6* promoter that lacks a Pitx2 binding site (Fig 12C). The enrichment of chromatin achieved using the Pitx2 antibody represented a 37-fold increase (Fig 12D; comparison is to control IgG). A luciferase reporter assay using a construct bearing the *FoxO6* promoter (4Kb) showed that co-transfection of LS-8 cells with PITX2 led to an ~60-fold increase in reporter activity over transfection with the reporter construct alone (Fig 12E). This effect was specific, as shown using enhancer constructs containing either the intact 70-bp *FoxO6* PITX2 enhancer or the same site with a mutation in the PITX2 binding site (Mut enhancer) within the luciferase vector (has Thymidine Kinase (TK) minimal promoter). These enhancer constructs were co-transfected into LS-8 cells with the PITX2 cDNA or empty vector, and mutation of the PITX2 binding element abolished PITX2 activation (Fig 12F). We have recently shown that Pitx2 regulates Hippo signaling through a direct interaction with Yap to control heart regeneration [86]. These data demonstrate another role for Pitx2 regulating FoxO6, which activates Lats1 expression.

## Discussion

This report describes how restricted expression of *FoxO6* in the brain and craniofacial region activates Hippo signaling to regulate growth of the anterior part of the skull, brain, maxilla, mandible and teeth. Because Hippo signaling is ubiquitous in the developing embryo, specific factors must modulate its activity in a tissue- and organ-specific manner at particular points in development. Our MRI analyses have identified specific regions that are expanded in the *FoxO6*^*-/-*^ mice: the frontal cortex, the olfactory system, and the pituitary regions. It is notable that all of these regions are linked to growth of the frontonasal prominence. The unique expression pattern of FoxO6 appears to control the development and size of the craniofacial skeleton and anterior structures after birth.

The temporal, spatial and tissue-specific timing of expression of specific factors drives patterning and growth of the facial prominences. In humans, facial characteristics vary by ethic and cultural background. Individuals can present with maxillary and/or mandibular prognathism (increased growth of the upper and lower jaw, respectively), micrognathia (small jaw) or retrognathia (retracted jaw). Each of these conditions affects the development of the dentition demonstrating that odontogenesis and jaw growth are directly linked.

Early in development (E8.5 to E10.5 in mice), cranial neural crest (CNC) migrate to populate the frontonasal prominence and the first pharyngeal arch [87–90]. Modulation of gene expression in the CNC, by numerous factors has been shown to guide craniofacial development [19, 42, 91, 92]. The head ectoderm contains cells and centers necessary for the early development, patterning and specification of the craniofacial skeleton. The epithelium of the frontonasal prominence and first pharyngeal arch express several signaling proteins that induce transcription factor expression in the neural crest-derived mesenchyme. For example, Wnt signaling in the frontonasal process induces growth and morphogenesis [21, 31, 93–105], and Shh expression in the facial ectoderm regulates morphogenesis of the head and face as well as development of the maxillary process [21, 106]. These molecular mechanisms all drive early patterning (initial patterning and segregation of tissues) within the craniofacial region. However, little is known about factors that influence later stages in the development of facial morphology, *FoxO6* is a good candidate, because it is expressed at later embryonic stages and does not appear to affect cell and tissue-specific patterning.

The evolution of the mammalian skull and its morphologically complex traits has been extensively studied as the development of three distinct modules: the calvarium, the cranial base, and the face [25, 107–109]. The hypothesis that growth of the brain and face are linked is supported by some evidence from mouse models [25, 26, 110, 111]. This hypothesis is strengthened by work showing that development of the cerebellar structure is associated with isolated cleft lip and/or palate [112]. The face forms from the maxillary, mandibular and frontonasal prominences and a complex gene regulatory network that tightly controls development of these tissues [21, 113, 114]. As the brain and skull grow, the facial prominences also grow and both converge to form the face. The rate of growth of each can influence outgrowth of the midface and produce either prognathism or retrognathism. An elegant study demonstrated that forebrain and facial shape differ between mouse strains [25]. Although the variations noted in that study were limited to developmental stages with complex developmental processes and molecular mechanisms that remain undefined, it is clear from that study that epigenetic integration and sensitivity to gene dosage can affect facial morphology [25, 26, 44, 115]. Growth of the craniofacial complex (skull and brain) involves the integration of signaling pathways that pattern both during development [116].

Crosstalk between the molecular components of the TGF-β, WNT, Notch, Hedgehog, Fgf and Hippo pathways influences the control of both development and cell proliferation [2, 117–139]. Interacting components of one such set of pathways are Smad (of the TGF-β pathway) and β-catenin (of the WNT pathway): both accumulate in the nucleus to activate shared targets, and interact with Lef/TCF transcription factors in inducing gene expression and controlling cell fate [124, 125, 140]. Hippo is another interface for these two pathways; its activation results in cytoplasmic retention of TAZ/YAP proteins, which can also interact with Smads to inhibit their activity, and thus Hippo activity antagonizes WNT signaling [2, 135–137, 141]. TAZ/YAP can also interact with β-catenin to activate gene expression during development [2, 142, 143]. Pitx2 is the earliest transcriptional marker of tooth development, and interacts with β-catenin to regulate gene expression [77, 81, 82, 144, 145]. Pitx2 was identified as a potential modulator of craniofacial morphogenesis involving ephrin-B1 signaling and cell proliferation [34]. Given that this protein is expressed in both the brain and craniofacial regions, it may link brain and craniofacial development by activating FoxO6.

Hippo signaling regulates *Runx2* expression, and *FoxO6* loss-of-function mice have decreased levels of Runx2, a protein that can interact with TAZ/YAP to activate gene expression. Several studies have demonstrated a role for Runx2 in modulating facial size, and length [146–148]. Mutations and polymorphisms contribute to variations in facial morphology and evolution of face shape changes in carnivoran species [149]. Thus, FoxO6 may directly regulate Runx2 independent of Hippo signaling to delay osteogenesis in relation to increased skeletal growth. However, the decrease in *Lats1* expression in the *FoxO6*^*-/-*^ mouse would lead to activation of cell proliferation by promoting the nuclear accumulation of TAZ/YAP and an increase in the expression of WNT/β-catenin target genes. Lats1/2 phosphorylates YAP and TAZ, leading to the retention of these proteins in the cytoplasm and preventing them from activating gene expression. TAZ inhibits Wnt signaling by suppressing DVL2 and preventing β-catenin from entering the nucleus to stimulate gene expression [2]. Furthermore, TAZ/YAP and TGF-β/Smads can interact with β-catenin in the nucleus, thereby activating a gene regulatory network that controls cell proliferation and organ size. Thus, the direct regulation of *Lats1* by FoxO6 is a new mechanism for controlling craniofacial growth, by inhibiting the cell proliferation controlled by the Hippo signaling pathway.

In *FoxO6*^*-/-*^ mice, the incisors had low *Lats1/2* expression compared with control mice and were expanded in length and size. During development of the mouse incisor, the stem cells proliferate (LaCL) and populate the transient amplifying (TA) zone of the inner enamel epithelium (IEE) and migrate to differentiate into ameloblasts that secret enamel [150]. We conclude that the increased *amelogenin* expression in *FoxO6*^*-/-*^ mice may be caused by the observed increase in proliferation of the dental stem cells, as shown by *Ki67* staining and BrdU labeling. However, *Pitx2* expression was not changed and *Sox2*, a marker of dental epithelial stem cells [71, 150, 151], was slightly up-regulated in *FoxO6*^*-/-*^ embryos. The increase in incisor size correlates with an increase in the size of the anterior region of the mandible, whereas size of the molar was not affected. Dental patterning and tooth number were also not affected, suggesting that FoxO6-mediated regulation of Hippo signaling occurred later in development, after tooth development was initiated. However, we report a defect in pre-ameloblast polarization and differentiation, and this correlates with the role of Hippo signaling in the regulation of cell polarity [152]. A previous study of YAP over-expression in mice reported that the enamel organ was deformed and the dental lamina was widened [153], but that in the dental epithelium, proliferation was only slightly reduced, Shh, Fgf and Wnt levels were decreased, and epithelial cell movement and/or polarization was defective [153]. Interestingly, integration of the Hedgehog and Hippo signaling pathways is important in the proliferation of stem cells of Drosophila ovarian follicles, where Shh induces the expression of a transcriptional coactivator of the Hippo pathway [154]. Because Hippo signaling regulates stem-cell proliferation, self-renewal and differentiation, we speculate that crosstalk between the FoxO6, Hippo and Shh pathways may contribute to regulation of the proliferation and maintenance of dental stem cells [155].

The human face has many complex geometric variations. GM methods applied to both two and three dimensional craniofacial images, allow the exploration of specific patterns of craniofacial shape [156] that could be associated with genetic variation. There are three SNPs with high association results are known eQTLs for *FOXO6*. In addition, Pitx2 has been linked to Hippo signaling through its interaction with Yap [86] and our results show another role for Pitx2 in Hippo signaling through its regulation of *FoxO6* expression. Our results particularly support a role for *FOXO6* in variation related to horizontal projection of maxillary and mandibular structures. These features partly resemble those of the *FoxO6*^*-/-*^ mice. The activation of the Hippo pathway would impede growth in these specific regions and cause maxillary retrusion. Thus, mutations in *FOXO6* may define subtle shape alteration patterns that control the anterior growth of the human face.

**Fig 13:**
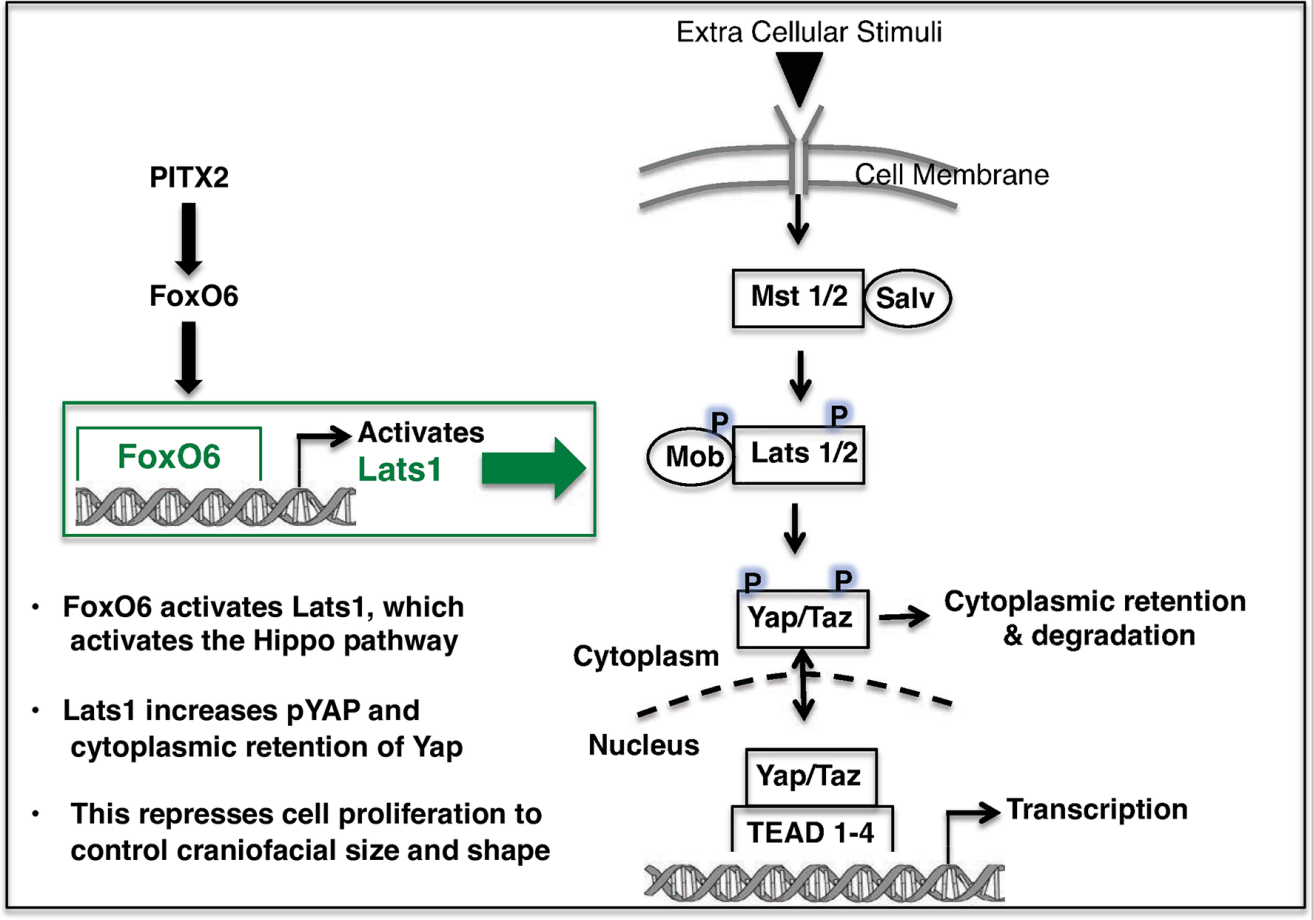
Model for *FOXO6* regulation of Hippo signaling in controlling growth of the head and craniofacial features. Mammalian Ste20 family kinases *Mst1* and *Mst2* form an active complex with Salvador (*Salv*), to further phosphorylate large tumor suppressor homolog (*Lats1* and *Lats2*) kinase. *Lats1* and *Lats2* bind to a scaffold protein, *Mob*, to further phosphorylate two transcriptional co-activator *Yap* and *Taz*. *Yap* and *Taz* are downstream Hippo signaling components and modulate the expression of genes involved in cell growth, proliferation and apoptosis. Phosphorylated Yap and Taz are retained in the cytoplasm. Unphosphorylated Yap and Taz can enter the nucleus to activate gene expression in concert with TEAD, P73, Pax3, Runx2 transcription factors and Smad and β-catenin. Pitx2 activates FoxO6 to maintain FoxO6 expression in craniofacial tissues. In this paper, we conclude that FoxO6 binds and activates *Lats1* to further inhibit Yap and cell proliferation, thereby controlling head and craniofacial growth regionally.

This study of the *FoxO6*^*-/-*^ mouse revealed increases in growth of the cerebral cortex and the incisors in the anterior parts of the face, with an overall increase in the length of the maxilla and mandible. These data suggest that FoxO6 controls the anterior-posterior growth of the craniofacial skeleton, and that differences in *FoxO6* expression my account for the differences in face morphology seen among mammals and other vertebrates. Furthermore, because FoxO6 regulates *Lats1/2* to control Hippo signaling independent of ligands or stimuli, this level of regulation may play important roles in other cell processes. A model for the role of FoxO6 is shown (Fig 13). We are currently exploring other transcriptional interactions and regulators of Hippo signaling during development.

## Materials and methods

### Ethics statement

"Institutional ethics (IRB) approval was obtained at each recruitment site and all subjects gave their written informed consent prior to participation (University of Pittsburgh Institutional Review Board #PRO09060553 and #RB0405013; UT Health Committee for the Protection of Human Subjects #HSC-DB-09-0508; Seattle Children’s Institutional Review Board #12107; University of Iowa Human Subjects Office/Institutional Review Board #200912764, #200710721 and #200811701). Animal care and use was approved by the University of Iowa Institutional Animal Care and Use Committee (IACUC), Protocol #1207146."

### Animals

All animals were housed at the University of Iowa, Program of Animal Resources and were handled in accordance with the principles and procedures specified in the Guide for the Care and Use of Laboratory Animals. All experimental procedures were approved by the University of Iowa IACUC guidelines. *FoxO6* knockout mice were generated using a gene-trap strategy. In these mice 19 kb of *FoxO6* genomic DNA (a stretch that contains the two *FoxO6* exons) were replaced with a LacZ cassette and a neomycin cassette surrounded by loxP sites for future excision. Briefly, *FoxO6* knockout embryonic stem (ES) cells (derived from C57BL/6 mice) were purchased from Knockout Mouse Project (KOMP) (project number: VG12465) and injected into blastocysts (from BALB/cJ) by the Texas A&M University Institute for Genomic Medicine. In two chimeras generated from ES cell injection, the mutant allele was passed through the germ line, and these animals produced heterozygous progeny. K14-Pitx2 transgenic mice were previously reported [62]. Mice were maintained on a C57BL/6 background. Observation of a vaginal plug was counted as embryonic (E) day 0.5, and embryos were collected at E14.5, E16.5, E18.5, P0 and P1. Mice and embryos were genotyped based on PCR carried out on DNA extracted from tail biopsies (WT primers: sense: 5’ACCTCATCACCAAAGCCATC3’, antisense: 5’GTCACCCTACCAGACCTCCA3’; KO primers: sense: 5’CCTGCAGCCCCTAGATAACTT3’, antisense: 5’GGTTGCTGGCTTCGTGTGGTG3’).

### Histology and immunofluorescence assay

Mouse embryos or heads were dissected in phosphate-buffered saline (PBS). Embryos were fixed with 4% paraformaldehyde-PBS solution for 0.5–4 hours. Following fixation, samples were dehydrated through graded ethanol, embedded in paraffin wax and sectioned (7 μm). Standard Hematoxylin and Eosin was used to examine tissue morphology as previous described [157]. For immunofluorescence (IF) assays, slides were boiled in 10mM sodium citrate solution (pH 6.0) for 20 minutes for antigen retrieval. They were then incubated with 20% goat serum-PBST for 30 min at room temperature, and then with antibodies against *Ki67* (Abcam, 1:200), amelogenin (Santa Cruz, 1:200), Lats1 (Cell signaling, 1:200) and pYap (Cell signaling, 1:200) and Beta-galactosidase (Abcam, ab9361, 1:50) at 4 ^o^C overnight. The slides were treated with FITC (Alexa-488)- or Texas Red (Alexa-555)-conjugated Secondary antibody for 30 minutes at room temperature for detection (Invitrogen, 1:500). Nuclear counterstaining was performed using DAPI-containing mounting solution.

### Detection of β-galactosidase (LacZ) activity

Mouse heads were stained for β-galactosidase activity according to standard procedures [63]. Embryos (from E12.5-E16.5) or embryo heads (from E17.5 to postnatal stage) were fixed for 30–60 min at RT in 0.2% glutaraldehyde in PBS. Fixed embryos were washed three times (1M MgCl_2_, 0.5M NaH_2_PO_4_, 0.2% Nonidet P-40 and 0.01% sodium deoxycholate in PBS) and stained 24–48 hours at 37°C using standard staining solution (5 mM potassiumferricyanide, 5 mM potassium ferrocyanide, 0.1% X-gal in wash buffer). On the next day, the samples were rinsed in PBS, photographed, and post-fixed in 4% formaldehyde for 0.5–4 hours. The fixed samples were dehydrated in a graded series of ethanol solutions, embedded in paraffin wax and sectioned. Sections were cut at 12-μm thickness and lightly counterstained with eosin.

### DNA cloning, shRNA, cell culture, transient transfection, luciferase, beta-galactosidase assay and Western blotting

*FoxO6* expression plasmid was cloned into pcDNA-myc 3.1 vector (Invitrogen) using the following primers: 5'- GCCTACATACCTCGCTCTGC -3' and 5'- ATCATAAGCTTGATTGGAGTTGGGTGGCTTA -3'. A 4kb DNA fragment in which the Pitx2 binding site was incorporated upstream of the *FoxO6* gene was cloned into the pTK-Luc vector (Promega) using the following primers: 5'-GGAAGCAATTGAAGTGGCCTTAGAT -3' and 5'- CAGATCCCAGAGCCGCGC-3'. A 1.7kb DNA fragment in which the FoxO6 binding site was incorporated upstream of the *Lats1* gene was cloned after the luciferase gene in the pTK-Luc vector (Promega) using the following primers: 5'- TCAGTGGATCCAGATCCCCTGAAGCTGGAGT -3' and 5'-TGACTGGATCCCAACATTGGGCACTGACATT -3'. The *FoxO6* shRNA targets the 5’- CTCCAATCTGGTTCTCAAATGACAC -3’ sequence of *FoxO6* mRNA was cloned into pSilencer 4.1 (Life Technologies). The 5’-CCTAAGGTTAAGTCGCCCTCG-3’ sequence was cloned into pSilencer 4.1 to generate a scrambled shRNA control. The Hippo reporter constructs HOP-Flash (8x TEAD binding sites) and HIP-Flash (mutant TEAD binding sites) were obtained from Addgene [158].

CHO, LS-8 cells (oral epithelial-like cell), HEPM (ATCC, Human Embryonic Palatal Mesenchyme) and ET-16 cells (dental epithelial cell) were cultured in DMEM supplemented with 10% fetal bovine serum (FBS) and penicillin/streptomycin and were transfected by electroporation. Cultured cells were fed 24 h prior to transfection, resuspended in PBS and mixed with 5 μg expression plasmids, 10 μg reporter plasmid and 0.5 μg SV-40 ß-galactosidase plasmid. Electroporation of CHO, LS-8 cells and ET-16 cells was performed at 400 V and 750 microfarads (μF) (Gene Pulser XL, Bio-Rad). Transfected cells were incubated for 24–48 h in 60 mm culture dishes and fed with 10% FBS and DMEM and then lysed and assayed for reporter activity and protein content by Bradford assay (Bio-Rad). FOX06, shFOX06, YAP5SA, and YAP WT were transfected into CHO cells at differing ratios of cDNA/reporter/ß-gal using PEI. 20 h after transfection, cells were placed in low serum media (0.5%) and harvest in reporter lysis buffer 40h after transfection.

Luciferase was measured using reagents from Promega. β-galactosidase was measured using the Galacto-Light Plus reagents (Tropix Inc.). All luciferase activities were normalized to β-galactosidase activity. For Western blot assay, cell lysates (10 μg) were separated on a 10% SDS–polyacrylamide gel and the proteins were transferred to PVDF filters (Millipore), and immunoblotted using the following antibodies FoxO6, (Abcam, 1:1000), Lats1 (Cell signaling, 1:1000), Yap (Cell signaling, 1:1000), pYap (Cell signaling, 1:1000), PITX2 (Capra Sciences, 1:1000), GAPDH (Santa Cruz, 1:1000) or β-Tubulin (Santa Cruz, 1:2000). ECL reagents from GE HealthCare were used for detection.

### Chromatin immunoprecipitation assay (ChIP)

The ChIP assays were performed as previously described using the ChIP Assay Kit (Upstate) with the following modifications [159]. LS-8 cells were plated in 60 mm dishes and fed 24 h prior to the experiment, harvested and plated in 60 mm dishes. Cells were cross-linked with 1% formaldehyde for 10 min at 37°C. Cross-linked cells were sonicated three times to shear the genomic DNA to DNA fragments average ranged between 200 and 1000 bp. DNA/protein complex were immunoprecipitated with specific antibody (Pitx2 antibody, Capra Sciences, PA-1023; FoxO6 antibody, Abcam, ab48730). DNAs from the precipitants were subject to PCR to evaluate the relative enrichment. The following primers were used to amplify the *FoxO6* promoter region, which contains a Pitx2 binding site: The sense primer (5- GGGGAGGAACTCACTCGTTT -3) and the antisense primer (5- GAGCTGCTCCCTTTGAGGTC -3). The following primers were used to amplify the *Lats1* promoter region, which contains a FoxO6 binding site: the sense primer (5- TTCCCAGCAGGACTCTGTCT -3) and the antisense primer (5- CAAATGCCACTTTCTGGTGA -3). All PCR reactions were done under an annealing temperature of 60°C. All the PCR products were evaluated on a 2% agarose gel for appropriate size and confirmed by sequencing. As controls the primers were used in PCRs without chromatin; normal rabbit IgG was used replacing the specific antibody to reveal nonspecific immunoprecipitation of the chromatin. 3 parallel Realtime PCRs were also performed in triplicates using these primers to quantify the enrichment of DNA pulled down by specific antibody over the DNA pulled down by IgG control. Primers designed according to 3.5 kbp upstream (Sense: 5- TGTGCTCCAGGACTCCTCTT -3, antisense: 5- GCCATGGTCTAGCTCTGTCC -3) of the *FoxO6* promoter region containing no Pitx2 binding sites were used as negative controls. Primers located 4.9 kb upstream (Sense: 5- ATGGATCTCTCTGGCATTGG -3, antisense: 5- TCCTAGGCAGAGGCAGGTAA -3) of the *Lats1* promoter region containing no FoxO6 binding sites were used as negative controls.

### BrdU labeling

BrdU was injected into the pregnant mice (10μl/g of body weight, Invitrogen, 00–0103) 2 h prior to harvesting of E17.5 embryos. BrdU was directly injected into P7 mice 2 hour prior to harvesting, heads were decalcified and sectioned for staining. Embryos were embedded and sectioned as described previously [157], and sections were mounted and rehydrated with sequential concentrations of alcohol, followed by immersion in 3% H_2_O_2_ to block the endogenous peroxidase activity. Antigen retrieval was carried out by treating sections with 10 mM Sodium Citrate solution for 15 min at a slow boiling state. Sections were hydrolyzed for 30 min in 2 N HCl, neutralized for 10 min in 0.1 M sodium borate (pH 8.5), rinsed, blocked for 1 h in 10% goat serum, and immunostained with rat anti-BrdU antibody (1:250, ab6326, Abcam). *FoxO6*^*-/-*^ mice and WT sections were placed on the same slide and processed together for identical time periods.

### Cartilage and bone staining

For staining and visualization of whole skeletons, mice were dissected and skeletons were stained with alizarin red S and alcian blue 8G (Sigma), as previously described [160].

### Microarray and quantitative real time PCR gene expression analysis

Gene expression DNA microarray analyses were performed by LC Sciences (Houston, TX) using GeneChip Mouse Genome Mouse 430.2.0 Arrays. Total RNA was extracted from mandible and maxilla tissue of WT and *FoxO6*^*-/-*^ embryos using RNeasy Mini Kit from Qiagen (4 biological replicates were combined). Reverse transcription was performed according to the manufacturer's instruction (BIO-RAD iScript Select cDNA Synthesis Kit) using oligo (dT) primers. cDNAs were adjusted to equal levels by PCR amplification with primers to beta-actin (primers for beta-actin are 5'- GCCTTCCTTCTTGGGTATG-3’ and 5'- ACCACCAGACAGCACTGTG-3'). Primers for *FoxO6* q-PCR are: 5'- AAGAGCTCCCGACGGAAC -3' and 5'- GGGGTCTTGCCTGTCTTTC -3'. Primers for *Lats1* q-PCR are: 5'- TAGAATGGGCATCTTTCCTGA-3' TGCTATCTTGCCGTGGGT. Primers for *Lats2* q-PCR are: 5'-GACGATGTTTCCAACTGTCGCTGTG-3’ and 5'- CAACCAGCATCTCAAAGAGAATCACAC-3'. Primers for detecting *Runx2*, *Shh*, *Amelogenin*, *CCD1* and *Sox2* were previously described [65]. All of the PCR products were sequenced to verify that the correct band was amplified.

### MRI methods

Magnetic resonance imaging (MRI) was performed on a 4.7-T Varian small-bore scanner. All acquisitions utilized a 25-mm diameter transmit/receive coil for high-resolution imaging. Mice were anesthetized with isoflurane (3% induction, 1.5% maintenance) and transferred to the scanner for imaging. After a series of three-localizer scans (each about 5 sec long), a set of T2-weighted fast spin-echo images was acquired in the axial plane. The protocol parameters were TR/TE. 2,100/60 msec, echo train length of eight, 0.5-mm thick contiguous slices with in-plane resolution of 0.16mm X 0.16mm over a 256 X 256 matrix using 12 signal averages. The total time for the entire protocol was about 40 min. All MRI data were processed using BRAINS software developed locally at the University of Iowa [161]. The mouse brain atlas used for segmentation purposes was the mouse Biomedical Informatics Research Network (mBIRN) atlas, which was constructed using T2-weighted magnetic resonance microscopy (MRM) from 11WTC57BL/6J mice at the University of California, Los Angeles [162].

For our pipeline process we employed a directed acyclic pipeline architecture using Nipype [163], a Pythonbased wrapping library for neuroimaging applications. ThemBRIN atlas was registered to the input T1 file using b-spline warping within BRAINSFit [164], a mutual information driven application developed under the ITK framework. The atlas was then resampled using BRAINSResample to match the voxel lattice of the T1 image, thereby allowing one-to-one correspondence between atlas and image. Finally, we computed the volume measurements for each desired region of our atlas as the sum of voxels within a given label times the volume of a voxel. The atlas defined 43 regions of interest. The regions were then grouped into the following areas: amygdala, hypothalamus, pituitary, thalamus, total brain volume, basal ganglia, brainstem, cerebrospinal fluid (CSF), cerebellum, hippocampus, white matter tracts, anterior cortex, and posterior cortex. The anterior cortex was further subdivided into the olfactory and frontal cortices. The posterior cortex was similarly subdivided into the posterior, entorhinal, and perirhinal cortices. Volumes were reported in mm3. All analyses were performed using Statistical Package for Social Science (SPSS), version 19.0 for Windows (SPSS, Inc., Chicago, IL). Due to the small sample size, non-parametric analysis using the rank of all measures was utilized in order to minimize any effects of outliers. Analysis of variance (ANOVA) was used to evaluate total brain volume across groups. The remainder of the brain regions was compared across the two groups using Analysis of Covariance (ANCOVA), controlling for total brain volume.

### Imaging and Microcomputed tomography (microCT)

To analyze variation in gross craniomandibular dimensions, we imaged n = 3 WT and n = 2 *Fox06*^-/-^ mice using a Siemens Inveon Micro-CT/PET scanner. Skulls were scanned at 60kVp and 500mA with a voxel size of 30μm and reconstructed images were imported into Osirx DICOM imaging software [165] for morphometric analysis. Using two- and three- dimensional renderings we collected a series of linear anterior-posterior and transverse skeletal and dental measurements defined in Fig 3. Hemimandibles with soft tissues removed were scanned in a μCT-40 (Scanco, Brüttisellen, Switzerland) at 70 kV, 114 mA, and 6 μm resolution. Images were processed with μCT-40 evaluation software and FIJI (https://fiji.sc/) was used to orient the mandibles in a standardized way based on anatomical landmarks to clearly observe and compare enamel mineralization in two planes 1) through the first molar, in a coronal plane through the distal root and, extending this plane, in the early maturation stage of the developing incisor and, 2) in the maturation stage incisor, in coronal plane through the mandible.

### Statistical analysis for experiments and bioinformatic analyses

For each condition, three experiments were performed with results are presented as the mean ± SEM. The differences between two groups of conditions were analyzed using an independent, two-tailed t-test.

Gene expression data were normalized by MAS5 algorithms. Differential expression genes were identified using Limma package in R. To identify altered pathways in *FoxO6* knockout mice. We ran Gene Set Enrichment Analysis (GSEA) with Broad Institute GSEA software (GSEA Preranked command line with default parameters) [166]. We ranked all genes based on its fold change between *FoxO6* knockout and wild type mandible and maxilla tissue. P value was calculated by permutation test. P value was adjusted by Benjamini & Hochberg's False Discovery Rate (FDR). RNA was pooled from 4 biological replicates for WT and *FoxO6* mutant mice including null and heterozygotes (RIN numbers were >9 for the samples).

## Supporting information

S1 Table Measurements and % change between WT and *FoxO6*^*-/-*^ mice. WT and *FoxO6*^*-/-*^ mice were analyzed for growth by uCT analyses. The percent (%) increase and p values are show for each measurement.
(PPTX)

S1 Fig Specific genes and processes regulated by FoxO6. WT and *FoxO6*^*-/-*^ mice mandibles were harvested and RNA isolated and gene expression probed using RNA-sequencing. **A)** A heat map from the RNA-seq data showing that *Lats1* and *Lats2* are decreased in the *FoxO6* null mice. **B)** A gene ontology map showing that FoxO6 regulates transcription and cell signaling.
(TIF)

S2 Fig FoxO6 immunostaining and expression in the mandible and activation of Hippo signaling in cells. **A-D)** A FoxO6 antibody was used to demonstrate FoxO6 expression by immunostaining in WT E16.5 mandibles and lower incisors. FoxO6 is highly expressed in the oral epithelium (OE), dental lamina (DL), and dental epithelium of the lower incisor, but not in *FoxO6*^*-/-*^ embryos. The expression domains of FoxO6 correlate with X-gal staining. **E)** FoxO6 transcripts are absent from the *FoxO6*^*-/-*^ E16.5 embryos. **F)** FoxO6 activation of Hippo signaling was accessed by transfection of FoxO6, shFoxO6, Yap 5SA and Yap with the HOP and HIP luciferase reporter constructs. FoxO6 decreased HOP activation in a dose dependent response, while knockdown of endogenous FoxO6 (shFoxO6) activated HOP luciferase expression in a dose dependent response. Yap 5SA served as a positive control to demonstrate the HOP reporter was active. **p<0.01.
(TIF)

S3 Fig FoxO6 regulates dental epithelial cell proliferation in older mice and in cell-based experiments. **A,B)** Cell proliferation in P7 WT and *FoxO6*^*-/-*^ mice, as assessed by BrdU injection (2 hours prior to sacrifice), respectively. The white line shows the outlines the transit amplifying cells undergoing proliferation in the *FoxO6*^*-/-*^ mice. Scale bar represents 100μm. **C)** Quantitation of the BrdU-positive cells in sections of lower incisors. **D)** CHO cells were transfected with either FoxO6, shFoxO6 (inhibits FoxO6 endogenous expression) or empty vector plasmid DNA and cell proliferation was determined ever 24 hours using the MTT assay.
(TIF)
