# Supplementary material for: FoxO6 regulates Hippo signaling and growth of the craniofacial complex
Source: PLoS Genet. 2018 Oct 4;14(10):e1007675. doi: 10.1371/journal.pgen.1007675 (PMC6197693; doi:10.1371/journal.pgen.1007675)
Supplement: S1 Table — WT and FoxO6-/- mice were analyzed for growth by uCT analyses. The percent (%) increase and p values are show for each measurement. (PPTX) [file pgen.1007675.s001.pptx]

## Slide 1
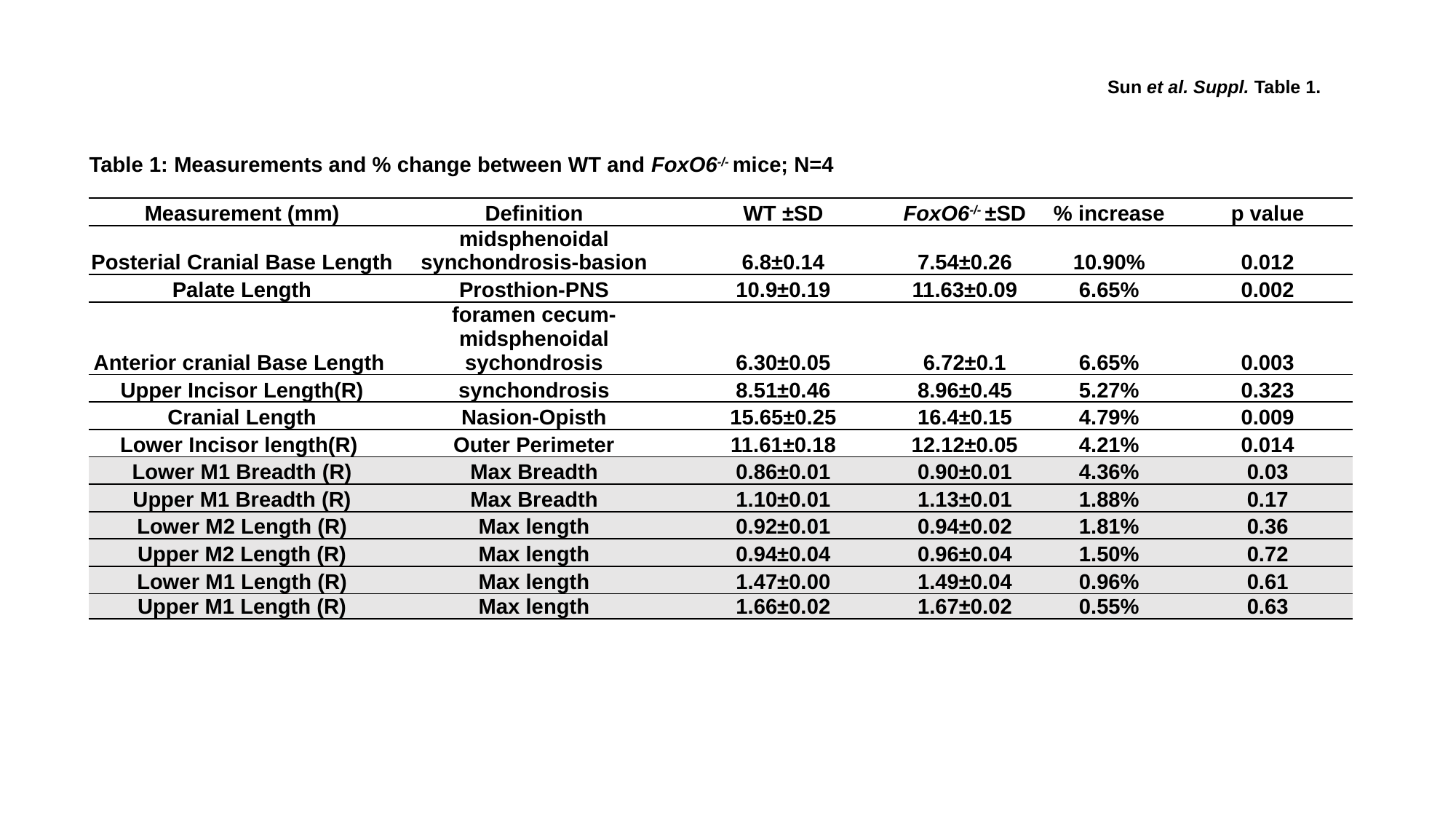

Sun et al. Suppl. Table 1.
Table 1: Measurements and % change between WT and FoxO6-/- mice; N=4
| Measurement (mm) | Definition | WT ±SD | FoxO6-/- ±SD | % increase | p value |
| --- | --- | --- | --- | --- | --- |
| Posterial Cranial Base Length | midsphenoidal synchondrosis-basion | 6.8±0.14 | 7.54±0.26 | 10.90% | 0.012 |
| Palate Length | Prosthion-PNS | 10.9±0.19 | 11.63±0.09 | 6.65% | 0.002 |
| Anterior cranial Base Length | foramen cecum-midsphenoidal sychondrosis | 6.30±0.05 | 6.72±0.1 | 6.65% | 0.003 |
| Upper Incisor Length(R) | synchondrosis | 8.51±0.46 | 8.96±0.45 | 5.27% | 0.323 |
| Cranial Length | Nasion-Opisth | 15.65±0.25 | 16.4±0.15 | 4.79% | 0.009 |
| Lower Incisor length(R) | Outer Perimeter | 11.61±0.18 | 12.12±0.05 | 4.21% | 0.014 |
| Lower M1 Breadth (R) | Max Breadth | 0.86±0.01 | 0.90±0.01 | 4.36% | 0.03 |
| Upper M1 Breadth (R) | Max Breadth | 1.10±0.01 | 1.13±0.01 | 1.88% | 0.17 |
| Lower M2 Length (R) | Max length | 0.92±0.01 | 0.94±0.02 | 1.81% | 0.36 |
| Upper M2 Length (R) | Max length | 0.94±0.04 | 0.96±0.04 | 1.50% | 0.72 |
| Lower M1 Length (R) | Max length | 1.47±0.00 | 1.49±0.04 | 0.96% | 0.61 |
| Upper M1 Length (R) | Max length | 1.66±0.02 | 1.67±0.02 | 0.55% | 0.63 |
