# Supplementary figures and images for: FoxO6 regulates Hippo signaling and growth of the craniofacial complex
Source: PLoS Genet. 2018 Oct 4;14(10):e1007675. doi: 10.1371/journal.pgen.1007675 (PMC6197693; doi:10.1371/journal.pgen.1007675)

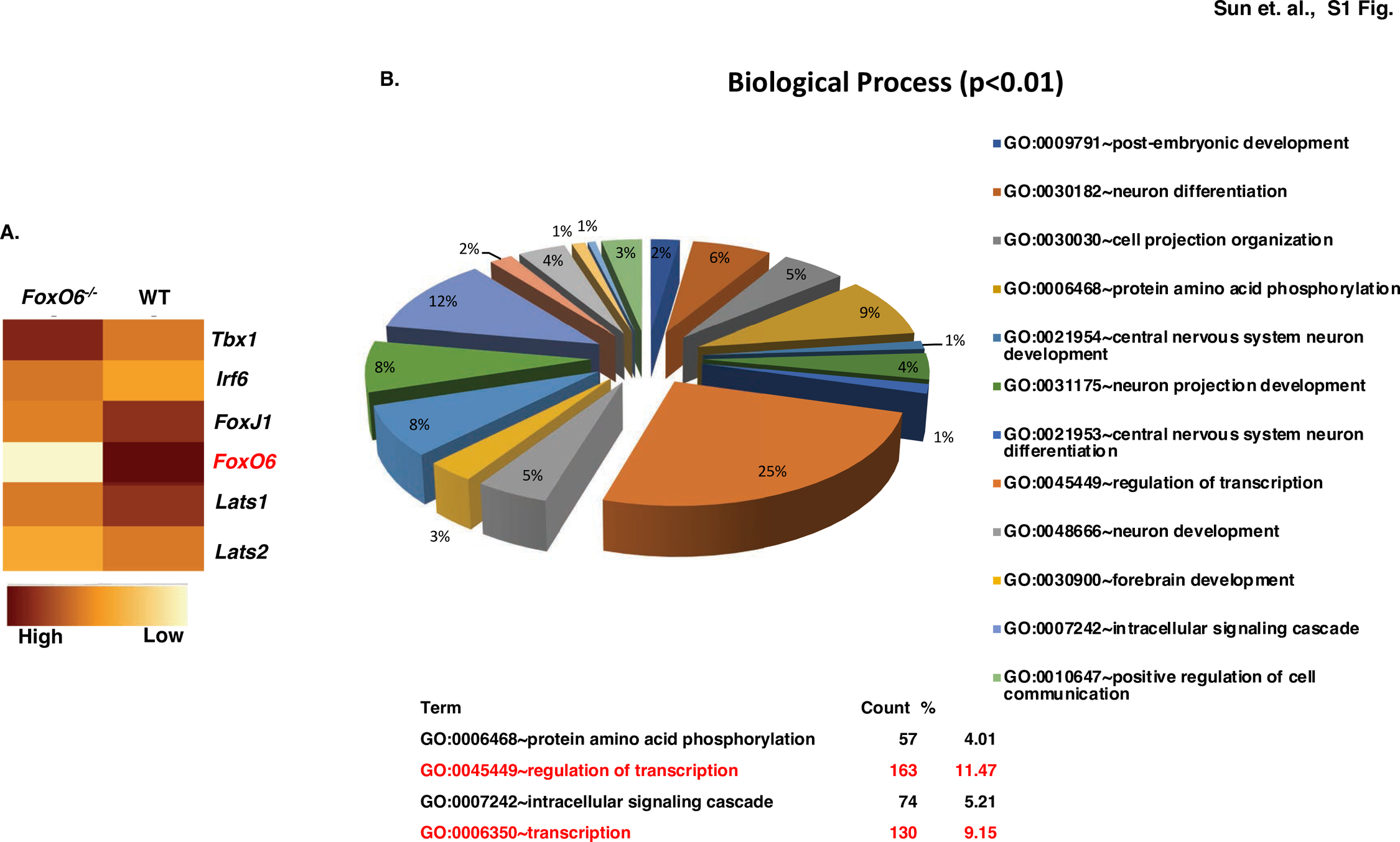

Supplement: S1 Fig — WT and FoxO6-/- mice mandibles were harvested and RNA isolated and gene expression probed using RNA-sequencing. A) A heat map from the RNA-seq data showing that Lats1 and Lats2 are decreased in the FoxO6 null mice. B) A gene ontology map showing that FoxO6 regulates transcription and cell signaling. (TIF) [file pgen.1007675.s002.tif]

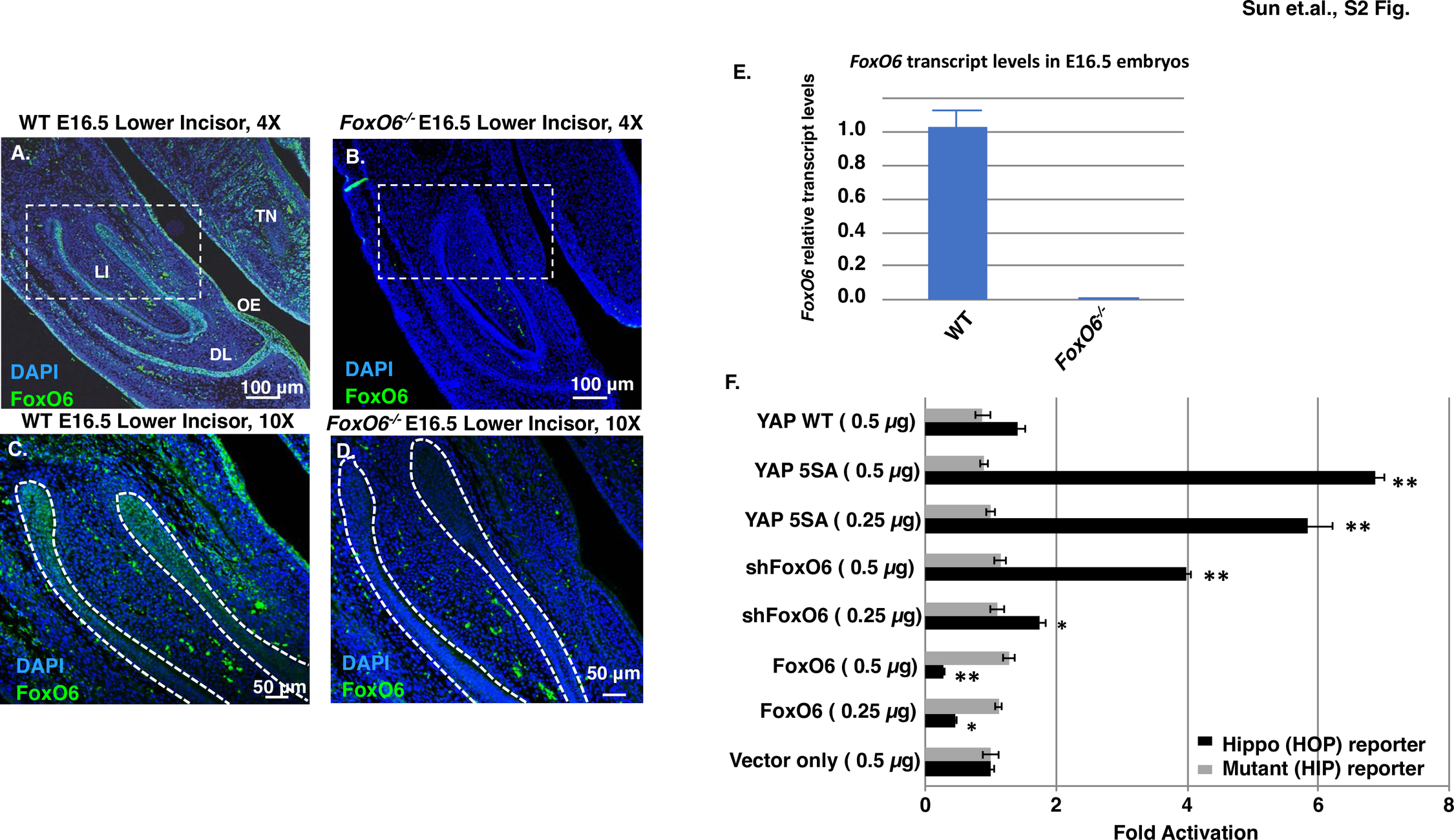

Supplement: S2 Fig — A-D) A FoxO6 antibody was used to demonstrate FoxO6 expression by immunostaining in WT E16.5 mandibles and lower incisors. FoxO6 is highly expressed in the oral epithelium (OE), dental lamina (DL), and dental epithelium of the lower incisor, but not in FoxO6-/- embryos. The expression domains of FoxO6 correlate with X-gal staining. E) FoxO6 transcripts are absent from the FoxO6-/- E16.5 embryos. F) FoxO6 activation of Hippo signaling was accessed by transfection of FoxO6, shFoxO6, Yap 5SA and Yap with the HOP and HIP luciferase reporter constructs. FoxO6 decreased HOP activation in a dose dependent response, while knockdown of endogenous FoxO6 (shFoxO6) activated HOP luciferase expression in a dose dependent response. Yap 5SA served as a positive control to demonstrate the HOP reporter was active. **p<0.01. (TIF) [file pgen.1007675.s003.tif]

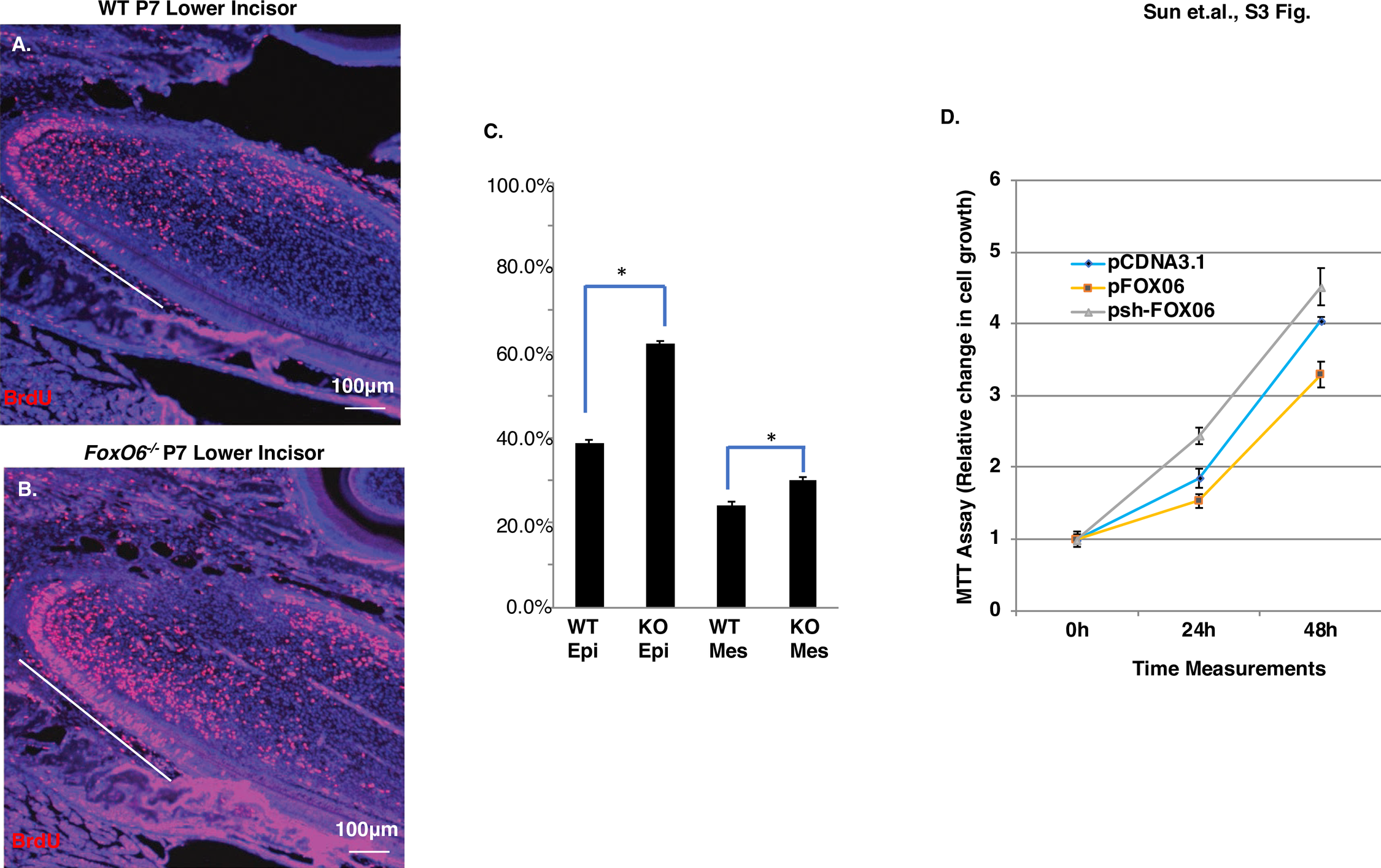

Supplement: S3 Fig — A,B) Cell proliferation in P7 WT and FoxO6-/- mice, as assessed by BrdU injection (2 hours prior to sacrifice), respectively. The white line shows the outlines the transit amplifying cells undergoing proliferation in the FoxO6-/- mice. Scale bar represents 100μm. C) Quantitation of the BrdU-positive cells in sections of lower incisors. D) CHO cells were transfected with either FoxO6, shFoxO6 (inhibits FoxO6 endogenous expression) or empty vector plasmid DNA and cell proliferation was determined ever 24 hours using the MTT assay. (TIF) [file pgen.1007675.s004.tif]
